# Development of Novel Therapeutics Targeting the Blood–Brain Barrier: From Barrier to Carrier

**DOI:** 10.1002/advs.202101090

**Published:** 2021-06-03

**Authors:** Jia Li, Meng Zheng, Olga Shimoni, William A. Banks, Ashley I. Bush, Jennifer R. Gamble, Bingyang Shi

**Affiliations:** ^1^ School of Pharmacy Henan University Kaifeng 475001 China; ^2^ Centre for Motor Neuron Disease Department of Biomedical Sciences Faculty of Medicine & Health Sciences Macquarie University Sydney New South Wales 2109 Australia; ^3^ Henan‐Macquarie University Joint Center for Biomedical Innovation School of Life Sciences Henan University Kaifeng Henan 475004 China; ^4^ Institute for Biomedical Materials and Devices School of Mathematical and Physical Sciences Faculty of Science University of Technology Sydney Sydney New South Wales 2007 Australia; ^5^ Geriatric Research Education and Clinical Center Veterans Affairs Puget Sound Health Care System and Division of Gerontology and Geriatric Medicine Department of Medicine University of Washington School of Medicine Seattle WA 98108 USA; ^6^ Melbourne Dementia Research Center The Florey Institute for Neuroscience and Mental Health The University of Melbourne Parkville Victoria 3052 Australia; ^7^ Center for the Endothelium Vascular Biology Program Centenary Institute The University of Sydney Sydney New South Wales 2042 Australia

**Keywords:** blood–brain barrier, brain diseases, drug delivery, nanomedicine, nanotechnology

## Abstract

The blood–brain barrier (BBB) is a highly specialized neurovascular unit, initially described as an intact barrier to prevent toxins, pathogens, and potentially harmful substances from entering the brain. An intact BBB is also critical for the maintenance of normal neuronal function. In cerebral vascular diseases and neurological disorders, the BBB can be disrupted, contributing to disease progression. While restoration of BBB integrity serves as a robust biomarker of better clinical outcomes, the restrictive nature of the intact BBB presents a major hurdle for delivery of therapeutics into the brain. Recent studies show that the BBB is actively engaged in crosstalk between neuronal and the circulatory systems, which defines another important role of the BBB: as an interfacing conduit that mediates communication between two sides of the BBB. This role has been subject to extensive investigation for brain‐targeted drug delivery and shows promising results. The dual roles of the BBB make it a unique target for drug development. Here, recent developments and novel strategies to target the BBB for therapeutic purposes are reviewed, from both barrier and carrier perspectives.

## Graphical abstract

Blood–brain barrier (BBB), which separates the blood and the brain, plays a critical protective role in brain homeostasis. However, it is also a formidable barrier for brain‐targeted drug delivery. This review summarizes the key strategies to restore BBB integrity and to penetrate the BBB for drug delivery, in particular, under neuroinflammation conditions.

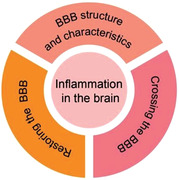

## 1 Introduction

**Figure 1:**
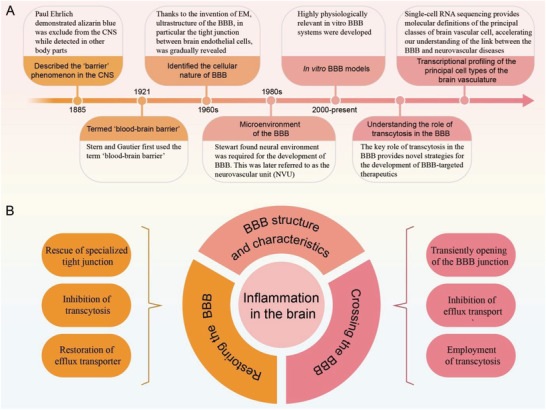
BBB structure and the development of therapeutics targeting the BBB. A) Historical timeline of key advances in understanding the BBB. B) The BBB is a key target for brain‐targeted therapeutics. 1) Disrupted BBB has been linked to the initiation and progression of neurological and cerebral vascular diseases. Restoration of BBB integrity may serve as a promising target for the treatment of these diseases. These strategies include the rescue of specialized tight junction in the brain endothelial cells, inhibition of transcytosis, and restoring efflux transporters. 2) The BBB remains a formidable obstacle to deliver drugs into the brain. Three major areas have been extensively explored to enhance brain‐targeted delivery: transiently opening of the BBB junction; inhibition of efflux transport and employment of transcytosis as a vehicle to facilitate transport. 3) Inflammation is a hallmark of brain diseases. Targeting inflammation restores the BBB, and BBB penetration is a prerequisite for therapeutics to fulfill potential in treating inflammation in the brain parenchyma. A combination of strategies in the restoration of the BBB and penetration of BBB are required to address the difficulties in the treatment of inflammation in the brain.

The existence of a special barrier in the brain was noticed in the late 19th century, as particular chemical dyes were excluded from the brain while staining other body tissues.^[^ ^1^ ^]^ In the early 1920s, this barrier was further defined as the blood–brain barrier (BBB).^[^ ^2^ ^]^ However, the structure of the BBB, including the specialized tight junctions, transcytosis, and efflux transporters, was not uncovered until the 1960s thanks to the development of new technologies such as electron microscope. The role of BBB microenvironment was later revealed,^[^ ^3^ ^]^ leading to a new concept of the BBB as the neurovascular unit (NVU) in 2001.^[^ ^4^ ^]^ More recently, emerging tissue engineering technologies provide highly physiologically relevant in vitro models that mimic the BBB. Despite problems with de‐differentiation, these models have significantly accelerated the study of the BBB.^[^ ^5^, ^6^, ^7^ ^]^ In the last decades, understanding the role and regulation of transcytosis has been significantly expanded. Several lines of evidence suggest that transcytosis plays an equally important role as that of tight junctions in the regulation of BBB integrity, leading to novel strategies for the regulation of BBB integrity and brain‐targeted drug delivery.^[^ ^8^, ^9^, ^10^ ^]^ Additionally, the advancement in next‐generation sequencing allows us to understand the BBB from a broad perspective at a whole transcriptome profiling level, thus offering a comprehensive data resource relevant to understanding the genetic and molecular underpinnings of BBB functions. These studies are likely to open a new chapter in the field of the BBB (**Figure** **1**A).^[^ ^11^, ^12^ ^]^

The maintenance of BBB integrity is essential for brain homeostasis and normal neurological function.^[^ ^3^, ^8^, ^13^, ^14^, ^15^, ^16^ ^]^ Loss of BBB integrity promotes the influx of toxins, plasma proteins, and immune cells from the blood, stimulating inflammation in the brain and subsequently leading to or exacerbating a range of cerebral vascular diseases and neurological disorders, such as ischemia/reperfusion stroke, hypertension, cerebral cavernous malformation, amyotrophic lateral sclerosis, Alzheimer's disease (AD), Parkinson's disease, multiple sclerosis, and depression.^[^ ^17^, ^18^, ^19^, ^20^, ^21^, ^22^, ^23^, ^24^ ^]^ Thus, restoration of BBB integrity presents a potential therapeutic target for treatment of these conditions.^[^ ^17^, ^25^, ^26^, ^27^, ^28^ ^]^ However, the highly restrictive nature of the BBB is also a major hurdle to the delivery of drugs to the brain, raising the possibility that restoration of BBB integrity may reduce therapeutic effectiveness by making it even harder for drugs to enter the brain.^[^ ^29^ ^]^ It is also the case, that despite a disrupted BBB existing in many neurological diseases, there is still limited penetration of even small molecules into brain parenchyma. Both lipophilic and hydrophilic small molecule therapeutics fail to appreciably cross the BBB to elicit a therapeutic effect in animal models of neurological disease.^[^ ^30^ ^]^ Similarly, in brain tumors, there are regions where the BBB remains intact enough to prevent drugs from reaching tumor cells at sufficient concentrations to fulfill therapeutic potential.^[^ ^31^ ^]^ It is interesting to note that recent studies suggest that the paracellular pathway is much less important than previously thought for therapeutics to reach tumor.^[^ ^32^ ^]^ Therefore, restoration of BBB integrity is highly unlikely to impede therapeutic entry into the brain parenchyma.

On the other hand, transient opening of the BBB, by means of physical force or chemicals, has been explored to enhance BBB penetration of drugs with promising results in animal studies and clinical trials.^[^ ^33^, ^34^, ^35^ ^]^ Evidence also suggests that the BBB is an active player to regulate the influx and efflux of molecules and cells between the brain and blood.^[^ ^36^ ^]^ This defines another important role of the BBB: as a transport carrier to mediate communication between two sides of the BBB. Several strategies, including using viruses, cells, or nanoparticles as vehicles, have been developed to transport substances of interest to the brain for therapeutic purposes. This field of investigation has shown tremendous progress in the past 30 years.

In this Review, we outline potential therapeutics targeting the BBB. We first summarize the structure and function of BBB to highlight its importance for brain function. We then discuss several key strategies to restore BBB integrity and summarize various approaches to deliver therapeutics across the BBB. Next, we take neuroinflammation as an example to demonstrate different strategies to develop targeted therapeutics: from both the barrier and carrier perspectives of the BBB. We end by highlighting the challenges of developing efficient BBB‐targeted therapeutics (Figure 1B).

## 2 Structure of BBB and the Role of Brain Endothelial Cells

**Figure 2:**
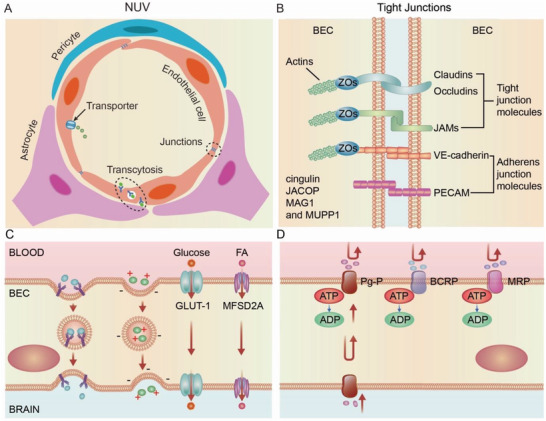
Neurovascular unit (NVU) structure and key features of brain endothelial cells (BECs). A) Cross‐sectional view of the NVU at the level of brain capillaries. Vessels are lined by a single layer of brain endothelial cells surrounded by astrocytes and pericytes. Three key features of brain endothelial cells include specialized tight junctions, low transcytosis, and high efflux transporter, which stringently coordinate and control the exchange of ions and nutrients between the blood and brain. B) Magnified view of the brain endothelial cell specialized tight junctions, which control paracellular permeability. Brain endothelial cells are tightly linked by tight junction molecules, including junctional adhesion molecules (JAMs), occludin, and members of the claudin family. Major adherens junction molecules include VE‐cadherin and PECAM‐1. The cytoplasmic adaptor proteins include zonula occludens (ZOs), cingulin, JACOP, MAG1, and MUPP1 link these transmembrane proteins to the cytoskeleton. C) Brain endothelial cells exhibit low levels of transcellular transport. Receptor‐mediated transcytosis (RMT); Adsorptive‐mediated transcytosis (AMT); Carrier‐mediated transport (CMT). D) Brain endothelial cells express efflux transporters that both shuttle specific nutrients into the brain and remove potentially harmful toxins and other small molecules from the brain, respectively.

The basic element of the mature BBB is the NVU (**Figure** **2**A).^[^ ^4^ ^]^ The NVU is mainly composed of brain endothelial cells and supporting cell structures such as the end‐feet of astrocytes, pericytes and may also include immune cells and neurons. Brain endothelial cells, surrounded by a specialized basal lamina and supporting cells, are the central anatomical and functional element of the NVU. In this review, we focus on brain endothelial cells. For an extended review of other components of the NVU, the reader can refer to other excellent reviews.^[^ ^4^, ^37^, ^38^, ^39^, ^40^ ^]^

Brain endothelial cells closely connect to each other to form a monolayer structure, the endothelium, producing the inner lining of cerebral blood vessels. Since the endothelium directly contacts the bloodstream, brain endothelial cells are at the frontline to control the exchange of compounds and cells between the blood and the brain parenchyma. Brain endothelial cells possess several special properties to fulfill the unique function of the BBB. Under physiological conditions, the junctions between brain endothelial cells are tightly sealed, allowing only small sized molecules (<400 Da) to cross the BBB passively.^[^ ^41^ ^]^ In addition, reduced vesicular transport and highly active efflux transporters on the brain endothelial cell surface facilitate only minimal but essential exchange. Recent studies attribute these properties of brain endothelial cells to a BBB‐specific gene expression profile, chromatin structure, and DNA methylation landscape.^[^ ^12^, ^42^ ^]^ In these studies, endothelial cells, isolated from brain and other peripheral organs such as heart, kidney, lung, and liver, were subjected to bulk and single cell RNA‐sequencing analysis. Compared with endothelial cells of peripheral organ origin, brain endothelial cells have enriched expression of genes related to tight junctions, transporters, extracellular matrix, and metabolic pathways. In addition, there is considerable heterogeneity in gene expression patterns along the brain vascular tree that consists of arteries, veins, and capillaries.^[^ ^11^, ^42^ ^]^ For instance, abundant expression of Mfsd2 and Tfrc genes is only observed in brain capillaries but not in arteries and veins. In contrast, Vwf and Vcam1 genes are more expressed in arteries and veins than in capillaries. Claudin‐5, the most enriched tight junction protein in brain endothelial cells, shows maximal expression in capillaries and small venules with minimal expression in larger venules.^[^ ^43^ ^]^ Despite the heterogeneity among different brain endothelial cells, three key features exist in all types of brain endothelial cells underlying the restrictive properties of the BBB: specialized tight junctions, low levels of transcytosis, and highly active efflux transporters.

### 2.1 Specialized Tight Junctions and Paracellular Transport

BBB properties are primarily determined by the extreme tightness of junctions between brain endothelial cells, leading to limited paracellular transit and low permeability (Figure 2B).^[^ ^41^ ^]^ The junctions between brain endothelial cells are mainly controlled by transmembrane proteins that include tight junction and adherens junction molecules.

Tight junction proteins include claudins, occludin, and JAMs. Claudin‐5 is the most abundant tight junction molecule in the BBB, although it is also seen in endothelial cells from other organs such as liver, lung, and kidney.^[^ ^44^ ^]^ Claudins‐1, ‐3, and ‐12 are also expressed by brain endothelial cells.^[^ ^45^ ^]^ Tight junction molecules form zipper‐like seals along the brain endothelial cell border, contributing to low paracellular permeability in the BBB. The major adherens junction molecule is VE‐cadherin but others include N‐cadherin and PECAM‐1, another junction protein that is luminally enriched.^[^ ^46^, ^47^ ^]^ Although adherens junctions are less prominent than tight junctions in the BBB, from a structural point of view, adherens junctions are formed at the early stages of intercellular contacts before the organized formation of tight junctions. Indeed, formation of tight junctions is dependent on adherens junction formation.^[^ ^48^, ^49^ ^]^ The mature BBB features close functional and physical crosstalk between tight junctions and adherens junctions.^[^ ^46^, ^50^ ^]^ For example, expression of tight junction molecule claudin‐5 is controlled by adherens junction molecule VE‐cadherin.^[^ ^51^ ^]^ In addition, transmembrane tight junction and adherens junction proteins connect, through their intracellular tails, to membrane‐associated cytoplasmic proteins and form a multimolecular complex. This complex further links to the actin cytoskeleton to control the tightness of the brain endothelium.^[^ ^46^, ^48^, ^50^ ^]^

### 2.2 Transcytosis and Other Transcellular Transport Routes

The low level of intracellular transport, including transcytosis and macropinocytosis has been well‐known as a key feature of the BBB.^[^ ^8^, ^16^, ^52^ ^]^ Transcytosis activity is higher in the brain endothelial cells during early development but is suppressed later, coinciding with the time course of BBB maturation.^[^ ^16^ ^]^ Pathologically, upregulated transcytosis, as shown by an elevated number of intracellular vesicles in the brain endothelial cells, has been implicated as an early and accurate indicator of BBB disruption.^[^ ^53^, ^54^ ^]^

Transcytosis is an apical‐to‐basolateral vesicular dependent intracellular transport mechanism. Compared to specialized tight junctions, which restrict paracellular transit, transcytosis in brain endothelial cells is partly responsible for transporting several large molecules, such as fatty acids and transferrin across the BBB.^[^ ^8^, ^55^ ^]^ Macropinocytosis, which is nonspecific, accounts for a large amount of protein transfer in peripheral capillary beds and is responsible for molecular weight‐independent leakage in the BBB.^[^ ^52^ ^]^ Although it was originally thought that the permeability of the BBB was mostly mediated by changes in tight junctions, recent evidence in mice and zebrafish suggests that transcytosis is equally important in the regulation of BBB integrity.^[^ ^8^ ^]^

In brain endothelial cells, transcytosis occurs for some macromolecules. In comparison, cell transport occurs by diapedesis, which only superficially resembles transcytosis; although these probably share some cellular mechanisms.^[^ ^56^, ^57^, ^58^ ^]^ There are 3 major steps in transcytosis, including endocytosis, intracellular vesicular trafficking, and exocytosis. Macromolecules first bind to brain endothelial cells through either an adsorptive‐mediated, carrier‐mediated, or receptor‐mediated processes (Figure 2C). AMT facilitates the passage of cationic molecules, such as cationic lipids, cationized albumin, and highly charged peptides, which interact with anionic microdomains on the brain endothelial cell cytoplasmic membrane through electrostatic interaction.^[^ ^59^, ^60^ ^]^ RMT depends on the expression of relevant receptors, which are highly selective for their ligands, on the surface of brain endothelial cells. For example, transferrin receptors are one of the most abundant receptors in brain endothelial cells and responsible for the transport of transferrin. Additionally, low‐density lipoprotein receptor‐related protein (e.g., LRP1, LPR8) transport low‐density lipoprotein and melanotransferrin across brain endothelial cells,^[^ ^61^ ^]^ whereas high‐density lipoprotein is mostly transported across brain endothelial cells through scavenger receptor BI (SR‐BI) mediated transcytosis.^[^ ^62^, ^63^ ^]^ There are several other types of transporters in brain endothelial cells, which are responsible for the transcellular movement of different substances, such as glucose, some non‐essential amino acids, fatty acids, and several ions, into the brain through pores and channels (CMT).^[^ ^64^, ^65^ ^]^

After binding to brain endothelial cells, macromolecules are then taken into the luminal surface of brain endothelial cells through internalization (termed endocytosis), generating small (60–120 nm) membrane vesicles that contain macromolecules. According to the structure of the endocytic machinery, endocytosis is typically either clathrin‐mediated or caveolae‐mediated. Clathrin‐coated vesicles consist of clathrin protein together with more than 50 other cytosolic proteins.^[^ ^66^ ^]^ The clathrin‐dependent pathway internalizes most of the molecules or cells binding to receptors. In comparison, caveolae are formed by the assembly of caveolins, integral membrane proteins that bind directly to membrane cholesterol. The caveolae‐dependent pathway is responsible for the transport of albumin and immune cells such as leukocytes and lymphocytes into the brain.^[^ ^67^ ^]^ Within brain endothelial cells, vesicles containing macromolecules may be degraded by cells through a process called lysosomal degradation or are recycled back to the apical membrane on the bloodstream side. In the case of transcytosis, these vesicles exit brain endothelial cells from abluminal surface to the brain side through exocytosis. Despite recent progress, the detailed mechanisms of how transcytosis is regulated remain largely elusive.^[^ ^68^ ^]^

### 2.3 Efflux Transporters

Efflux transporters on the brain endothelial cells are responsible for preventing exogenous substances from entering the brain or pumping brain‐produced metabolites and toxins back to the systemic circulation.^[^ ^69^, ^70^, ^71^ ^]^ Efflux transporters are mainly ATP‐binding cassette transporters (ABC transporters; Figure 2D). The most common ABC transporters include multidrug resistance mutation 1(MDR1)/P‐glycoprotein (P‐gp), ABC transporters G2 (ABCG2)/breast cancer resistance protein (BCRP), and several members of the MDR transporter family. These transporters can mediate saturable efflux at the plasma membrane. If saturated or insufficiently expressed, they may also function as a secondary defense mechanism by mediating intracellular lysosomal drug sequestration, leading to subsequent barrier‐body formation and disposal via phagocytosis by neutrophils.^[^ ^72^ ^]^ Transporters for nutritional molecules and essential amino acids are generally expressed on both luminal and abluminal sides of brain endothelial cells, whereas ABC efflux transporters are concentrated on the luminal, blood‐facing, plasma membrane of brain endothelial cells. Lower‐than‐normal expression and/or activity of an efflux transporter may be harmful to the brain, leading to the build‐up of toxins and neurological disease.^[^ ^71^, ^73^ ^]^

## 3 Restoration of BBB Function as a Therapeutic Target

BBB impairment has been found in many diseases of the brain.^[^ ^17^, ^18^, ^19^, ^20^, ^21^, ^22^, ^23^, ^27^, ^34^, ^74^, ^75^, ^76^, ^77^, ^78^, ^79^ ^]^ In some cases, such as human cognitive dysfunction and AD, BBB breakdown can be one of the earliest pathophysiological events or a major risk factor in disease initiation and development.^[^ ^80^, ^81^, ^82^ ^]^ BBB breakdown is preventable or inherently reversible, which provides a promising therapeutic target for the treatment of these diseases.

The mechanisms underlying how BBB dysfunction affects onset and progression of disease are not fully understood. Disrupted BBB‐elicited infiltration of peripheral blood factors, such as albumin, globulin, fibrinogen, may make a critical contribution.^[^ ^76^ ^]^For example, in AD, leaky BBB increased the influx of albumin and immunoglobulins, contributing to the formation and progress of disease by concentrating in areas of amyloid‐positive vessels and plagues.^[^ ^76^ ^]^ Prothrombin and thrombin were also found to be increased in senile plaques, leading to pro‐inflammatory response and neuronal cell death. More recently, extravasation of fibrinogen across the leaky BBB and deposition in brain parenchyma was found to contribute to microglia‐mediated dendritic spines elimination^[^ ^83^ ^]^ and release of neurotoxic reactive oxygen species, leading to neuronal dysfunction and cognitive decline. In addition, blood‐borne immune and inflammatory cells have also been found to cross leaky BBB to mediate progression in neurodegenerative disease, including epilepsy,^[^ ^84^ ^]^ AD,^[^ ^85^ ^]^ and multiple sclerosis.^[^ ^86^ ^]^

BBB dysfunction can be either associated with the destruction of tight junctions to increase in paracellular permeability, or changes in transcytosis and efflux activity. In addition, alterations in other brain endothelial cell properties such as enzymatic and secretory properties may also alter transcellular permeability without an obvious change in the tight junctions. Disease states may feature one type of BBB dysfunction or several, although they may not happen simultaneously.^[^ ^54^ ^]^ As exemplified by ischemia/reperfusion stroke, cytoskeletal alterations occur 30–60 min after stroke, followed by increased transcytosis as early as 6 h after stroke. Degradation of tight junctions is not detectable until 48 h after stroke and angiogenesis induced vascular permeability is seen at around 7 days.^[^ ^54^ ^]^

In this section, we review the recent progress in identifying BBB restoration as a therapeutic target in three categories: tight junction, transcytosis, and efflux transport.

### 3.1 Targeting Paracellular Permeability: Tight Junctions and Beyond

**Figure 3:**
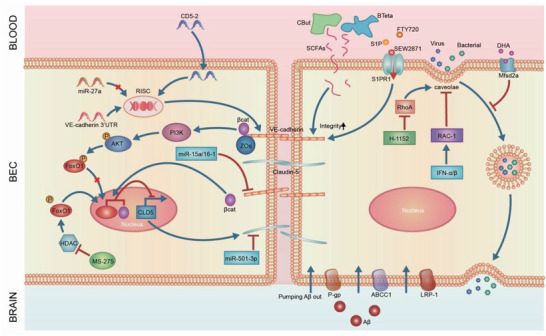
Restoration of BBB integrity as a therapeutic target. 1) Adherens and tight junction molecules. VE‐cadherin is the major adherens junction molecule. VE‐cadherin adhesion prevents FOXO1 accumulation in the nucleus to regulate claudin‐5 expression. Loss of VE‐cadherin leads to FOXO1 and *β*‐catenin translocation to nucleus, leading to repression of claudin‐5 transcription. CD5‐2, a target site blocker, binds to the binding site of miR‐27a to prevent its regulation of VE‐cadherin, leading to specific upregulation in VE‐cadherin expression and downstream claudin‐5. Claudin‐5 and ZO‐1 are major tight junction molecules. Deletion or inhibition of microRNAs, which target claudin‐5, restores the BBB. MS‐275, an inhibitor of HDAC, suppresses FOXO1 activity to rescue claudin‐5 expression. SCFAs, produced in the colon by bacteria, regulate occludin and claudin‐5 in the BBB in mice with depression. Treatment with bacteria such as *C. tyrobutyricum* (*CBut*) or *B. thetaiotaomicron* (*BTeta*) may serve as a new strategy to rescue BBB integrity. 2) Transcytosis. Bacterial and viral pathogens employ caveolae‐mediated transcytosis to escape lysosomal degradation and cross the BBB, leading to BBB breakdown. Inhibition of RhoA by its inhibitor H‐1152 or activation of Rac‐1 by interferons (IFN), including IFN‐*α* or IFN‐*β*, significantly reduced transcytosis across brain endothelial cells. Mfsd2a is a key molecule that inhibits transcytosis in the BBB. The lipid composition of brain endothelial cells and Msfd2a serve as key players in the regulation of transcytosis and may constitute targets to modulate transcytosis in the BBB for therapeutic purposes. 3) Efflux transporters. Lower efflux transporter expression and activity results in abnormal BBB function, contributing to neurological disease. In AD, LRP‐1 and ABC efflux transporter like P‐gp and ABCC1 are deficient, causing insufficient A*β* clearance and AD pathologies. Pharmacological rescue of these efflux transporters has been shown to alleviate AD in mice.

Under pathological conditions, junction molecules may change in expression level, phosphorylation status, or localization, resulting in disruption of junction integrity. By either limiting or reversing these events, inhibiting the paracellular permeability of the BBB and restoring its integrity can be achieved, leading to amelioration of disease symptoms. Thus, junction molecules, including adherens junctions and tight junctions, are proposed as therapeutic targets due to their critical roles in controlling paracellular permeability (**Table** **1** and **Figure** **3**).

**Table 1:** Summary of the targets and strategies to restore BBB integrity

	Therapeutic targets	Targeting strategies	Ref.
Paracellular permeability	VE‐cadherin	miR‐27a/VE‐cadherin interaction: e.g., BlockmiR CD5‐2	^[^ ^92^ ^]^
		S1PR1 agonist or modulator: e.g., SEW2871, FTY720	^[^^96^, ^97^, ^98^^]^
	Claudin‐5	Chronic antidepressant treatment: e.g., imipramine	^[^ ^21^ ^]^
		microRNAs: e.g., miR‐15a/16‐1	^[^ ^100^ ^]^
		Regulation of transcription factor: e.g., HADC inhibitor (MS‐275) for FOXO1; SOX18	^[^ ^17^ ^]^
		VE‐cadherin: e.g., BlockmiR CD5‐2	^[^ ^92^ ^]^
	ZO‐1	miR‐501‐3p	^[^ ^101^ ^]^
	Occludins	Short‐chain fatty acids (SCFAs)	^[^ ^102^ ^]^
Transcellular permeability	Caveolae‐mediated transcytosis	Small GTPase: RhoA inhibitor: e.g., H‐1152 Rac‐1 activator: e.g., IFN‐*α*, IFN‐*β*	^[^ ^103^ ^]^
		Msfd2a	^[^ ^15^ ^]^
Efflux transporter	P­gp	Pregnane X receptor (PXR) agonist	^[^ ^104^ ^]^
	ABCC1	Thiethylperazine	^[^ ^105^ ^]^
	Pharmacological restoration of LRP1 or GLUT1 expression	VEGF‐B inhibitor?	^[^ ^106^ ^]^

#### 3.1.1 Adherens Junctions

VE‐cadherin is the principal component of the adherens junction that plays a major role in regulating the integrity of vasculature. In normal vasculature, VE‐cadherin localizes at endothelial cell junctions. Loss of VE‐cadherin expression in junctions contributes to dysfunctional vascular integrity and disease development, including cerebral cavernous malformation, edema, diabetic eye complications, and solid tumor. In contrast, restoration of paracellular expression of VE‐cadherin or localizing VE‐cadherin in the junction areas is associated with junction repair, leading to disease regression.^[^ ^87^, ^88^, ^89^, ^90^, ^91^, ^92^ ^]^

There are limited methods to increase the expression of adherens junction genes in vivo. A microRNA, miR‐27a, has been shown to directly downregulate VE‐cadherin expression.^[^ ^93^ ^]^ CD5‐2, a target site blocker that specifically blocks miR‐27a/VE‐cadherin interaction, increases expression of VE‐cadherin.^[^ ^89^ ^]^ Treatment of cerebral cavernous malformation (a disease characterized by a disrupted BBB) model mice with CD5‐2 significantly reduced BBB permeability and the burden of cerebral cavernous malformation lesions.^[^ ^92^ ^]^

Localization of VE‐cadherin also affects BBB integrity.^[^ ^94^, ^95^ ^]^ Sphingosine‐1‐phosphate (S1P) is a bioactive sphingolipid. Through binding to its receptor, S1P receptor‐1 (S1PR1), S1P preserves the BBB by maintaining VE‐cadherin at the endothelial cell‐cell contact regions.^[^ ^94^ ^]^ Mice depleted of S1PR1 or S1P transporter apolipoprotein M (apoM) show increased flux of small molecules across the BBB, suggesting an increase in the BBB permeability. In contrast, systemic administration of a selective S1PR1 agonist, SEW2871, promptly reversed the increased paracellular BBB permeability in an apoM^–/–^ mouse model.^[^ ^96^, ^97^ ^]^ FTY720, a S1PR1 modulator, significantly prevented the intracellular redistribution of junction proteins and maintained BBB integrity, leading to amelioration of ischemia/reperfusion injury and neuroinflammation.^[^ ^98^ ^]^ This is of special interest since FTY720 was approved in the clinic as the first‐line oral drug for relapsing–remitting multiple sclerosis.^[^ ^99^ ^]^ It will be interesting to investigate whether its effect on the BBB has beneficial effects in other neurological diseases.

Since adherens junctions and tight junctions are functionally linked, modulation of VE‐cadherin may have enormous effects on the formation of tight junctions. VE‐cadherin upregulates the gene encoding the tight junction molecule claudin‐5 through AKT‐mediated forkhead box protein O1(FOXO1) phosphorylation and by limiting the translocation of *β*‐catenin to the nucleus of endothelial cells.^[^ ^51^ ^]^ Under disease conditions, such as excessive angiogenesis and inflammation, VE‐cadherin is absent or non‐functional, either due to phosphorylation or internalization. Consequently, FOXO1 and *β*‐catenin are accumulated in the nucleus, leading to inhibition of claudin‐5 expression and hence inducing BBB disruption. Since CD5‐2, a target site blocker of VE‐cadherin, significantly upregulates claudin‐5 in vitro and in vivo, it could have broad applications in treating neurological diseases associated with loss of claudin‐5 expression and tight junctions.

#### 3.1.2 Tight Junctions

Most studies focus on the key tight junctions in the BBB, the claudins (claudin‐1, ‐3, ‐5, and ‐12), and ZO. Decreased expression of claudin‐5 and ZO‐1 in brain endothelial cells has been linked to several neurological diseases. In depressed patient brains, which are associated with greater BBB permeability, expression of claudin‐5 is decreased.^[^ ^21^ ^]^ Genome‐wide studies in cognitively normal older individuals reveal a close link between claudin‐5 polymorphisms and cognition status.^[^ ^107^ ^]^ In multiple sclerosis and epilepsy, loss of claudin‐5 is an early and prominent feature.^[^ ^44^ ^]^ In a mouse model of depression, knockdown of claudin‐5 induces depression‐like behaviors, whereas chronic antidepressant treatment rescued claudin‐5 loss and promoted resilience.^[^ ^21^ ^]^ These results strongly suggest that restoration of tight junction could be a therapeutic target to restore BBB integrity and enhance disease treatment.

Unfortunately, to date, no molecules can directly increase the expression of tight junction molecules to restore BBB integrity. An alternative approach is to target regulators of tight junctions, such as microRNA or transcription factor.^[^ ^108^ ^]^ As an example, miR‐15a/16‐1 directly binds to claudin‐5 3′UTR to decrease its expression. Studies in genetically modified mice show that miR‐15a/16‐1 cluster haplodeficiency in endothelial cells enhances claudin‐5 expression, leading to amelioration of BBB dysfunction in mice with ischemic stroke.^[^ ^100^ ^]^ Additionally, microRNA miR‐501‐3p is directly bound to the 3′‐untranslated region of human ZO‐1 to decrease its expression.^[^ ^101^ ^]^ In vivo administration of a locked nucleic acid‐modified antisense oligonucleotide targeting miR‐501‐3p rescued ZO‐1 gene expression and BBB disruption in mice with vascular cognitive impairment, leading to significant rescue of cognitive impairment.^[^ ^101^ ^]^ Several transcription factors are involved in the regulation of claudin‐5 expression. FOXO1 is a negative regulator of claudin‐5 expression.^[^ ^51^ ^]^ Histone deacetylases (HDAC) are FOXO activators. Loss of HDACs prevents the nuclear accumulation of FOXO1, resulting in suppression of FOXO activity.^[^ ^109^ ^]^ Pharmacological inhibition of HDAC1 activity by MS‐275 (Entinostat) rescued claudin‐5 expression in the BBB and reduced depression‐like behavior in stress‐susceptible mice. This is consistent with transcriptomic analysis that identified HDAC1 as a mediator of stress susceptibility.^[^ ^17^ ^]^ In contrast to FOXO1, SRY‐box transcription factor 18 (SOX18) binds to a claudin‐5 promoter to activate claudin‐5 expression. SOX‐18 has been shown to promote barrier resistance of endothelial cells in vitro.^[^ ^110^, ^111^ ^]^ It will be interesting to evaluate if pharmacological stimulation of SOX18 expression can rescue the loss of claudin‐5 and restore BBB breakdown in vivo.

Although increasing claudin‐5 expression reduces BBB paracellular permeability and normalizes barrier function, the level of claudin‐5 must be stringently controlled. For example, in a murine model of AD, breaking down tight junctions by RNA interference (RNAi)‐mediated suppression of claudin‐5 and occludin allows for paracellular clearance of neurotoxic amyloid‐*β* (A*β*) peptides across the BBB, leading to enhanced clearance of neurotoxic A*β* from the brain to blood and improvement in cognitive function.^[^ ^79^ ^]^ This finding is seemingly counterintuitive as loss of claudin‐5 and tight junctions, partly via A*β*‐mediated downregulation, has been proposed to be a causal factor in AD.^[^ ^74^, ^80^, ^81^, ^112^, ^113^ ^]^ These apparent contradictions have raised a concern: should tight junctions be enhanced in the BBB? A previous study showed that claudin‐5 was responsible for the ability of the BBB to shield against the passage of molecules smaller than 800 Da.^[^ ^114^ ^]^ One possible explanation is that A*β*‐mediated and small interference RNA (siRNA)‐induced decreases in claudin‐5 enables the BBB to selectively pass non‐toxic low molecular weight A*β* monomer but not high molecular weight neurotoxic oligomers. Since A*β* oligomers are formed from assembly of A*β* monomer, lower levels of A*β* monomer reduce the chance to form neurotoxic A*β* oligomers. This suggests BBB permeability could be manipulated deliberately. However, controlled and targeted modulation of the BBB tight junctions requires a much deeper understanding before it can be considered for therapeutic purposes.

More recently, gut intestinal microbiota has been shown to directly regulate BBB permeability and function in both fetal and adult mouse brain.^[^ ^115^ ^]^ Indeed, intestinal microbiota has been linked, both in animal models and human patients, to neurological diseases, including AD,^[^ ^116^, ^117^ ^]^ autism,^[^ ^118^ ^]^ schizophrenia,^[^ ^119^ ^]^ and cerebral cavernous malformation.^[^ ^120^ ^]^ Germ‐free mice, which have never encountered a live bacterium, show increased BBB permeability compared to counterparts living in a normal environment. Short‐chain fatty acids (SCFAs), such as acetate, propionate, and butyrate, produced in the colon by bacterial fermentation of dietary fibers and resistant starch, have anti‐inflammatory functions.^[^ ^121^ ^]^ Treatment of germ‐free mice with bacteria such as *C. tyrobutyricum* (*CBut*) or *B. thetaiotaomicron* (*BTeta*) rescued BBB integrity by increasing expression of occludin in the frontal cortex and hippocampus.^[^ ^102^ ^]^

### 3.2 Targeting Transcellular Permeability: Transcytosis

The healthy BBB shows a low rate of transcytosis in brain endothelial cells, which is important in the maintenance of neurological function.^[^ ^8^ ^]^ One example is lipid receptor LRP1‐mediated transcytosis, which contributes to A*β* clearance. Studies in genetic modified mice with brain endothelial cell‐specific deletion of Lrp1 show reduced A*β* levels in the blood and elevated soluble A*β* in the brain, leading to deteriorations in spatial learning and memory.^[^ ^70^ ^]^ Knockdown expression of LRP1 with antisense RNA in mice shows similar results.^[^ ^122^ ^]^ Consistent with these results, in AD patients, LRP1 is also deficient in the BBB, contributing to slower clearance of A*β*.^[^ ^123^ ^]^ LRP1‐dependent A*β* clearance is regulated by glucose transport GLUT1.^[^ ^124^ ^]^ The expression of LRP1 and GLUT1 receptors on brain endothelial cells are closely correlated. Indeed, AD is also characterized by early reductions in glucose transport associated with diminished GLUT1 expression in brain endothelial cells, leading to BBB breakdown and cognitive dysfunction.^[^ ^125^ ^]^ Therefore, pharmacological restoration of LRP1 or GLUT1 expression may increase LRP‐1‐mediated efflux of A*β* clearance, representing a novel strategy for AD therapy. Recently, vascular endothelial growth factor (VEGF)‐B has been shown to inhibit cholesterol uptake and membrane cholesterol loading in endothelial cells, leading to a decrease in GLUT1‐dependent endothelial glucose uptake. Inhibiting VEGF‐B reconstitutes membrane cholesterol levels and restores glucose uptake.^[^ ^106^ ^]^ It will be interesting to evaluate if VEGF‐B inhibition via clinically approved drugs, such as simvastatin, can rescue transcytosis of A*β* clearance to promote AD treatment.

Additionally, pathogens can employ transcytosis in brain endothelial cells to cross the BBB and cause infection and brain neuroinflammation.^[^ ^126^ ^]^ For instance, several bacteria, such as *S. pneumoniae* and *E. coli*, enter the brain from the bloodstream even when the specialized tight junctions in brain endothelial cells remain intact, suggesting that these bacteria cross the BBB via transcytosis rather than passing through disrupted tight junctions.^[^ ^126^ ^]^ Although mechanisms that lead to transcytosis remain unclear, several lines of evidence suggest that bacteria bind to receptors, such as platelet‐activating factor receptor and polymeric immunoglobulin receptor, at the apical surface of brain endothelial cells, with subsequent invasion and translocation facilitated by clathrin‐dependent RMT.^[^ ^126^ ^]^ Viral pathogens can also employ caveolae‐mediated transcytosis to escape lysosomal degradation and cross the BBB, leading to neuroinflammation, as exemplified by Venezuelan (VEEV)and western equine encephalitis viruses (WEEV).^[^ ^127^ ^]^ These data indicate that inhibition of caveolae‐mediated transcytosis may maintain BBB integrity and prevent viruses entering the brain.

The Rho GTPases, Rho, and Rac, affect caveolae‐mediated transcytosis partly through remodeling of the actin cytoskeleton.^[^ ^128^, ^129^ ^]^ Inhibition of RhoA by its inhibitor H‐1152 significantly reduced VEEV transcytosis across brain endothelial cells.^[^ ^103^ ^]^ This is of special interest as Rho GTPases are viewed as good druggable targets with drugs already in the clinic. Additionally, activation of Rac‐1 by IFN, including IFN‐*α* and IFN‐*β*, also reduces caveolae‐mediated transcytosis in brain endothelial cells, preventing brain pathogen invasion.^[^ ^103^ ^]^

The lipid transporter, mfsd2a is another key regulator of caveolae formation and therefore caveolae‐mediated transcytosis.^[^ ^8^, ^15^ ^]^ Mfsd2a is specifically expressed at the luminal plasma membrane of brain endothelial cells to deliver the essential omega‐3 fatty acid docosahexaenoic acid across the BBB into the brain.^[^ ^64^ ^]^ Mice lacking mfsd2a present normal tight junctions while exhibiting increased levels of caveolae vesicles in brain endothelial cells, resulting in increased transcytosis and barrier permeability. Further studies show that lipids transported by mfsd2a create a unique lipid composition to specifically inhibit, caveolae‐mediated transcytosis, to maintain BBB integrity.^[^ ^15^ ^]^ In metastatic brain tumor, cancer cells break down the BBB through inhibition of mfsd2a expression, leading to enhanced transcytosis and alteration of lipid metabolism that facilitating metastasis.^[^ ^130^ ^]^ Therefore, both the lipid composition of brain endothelial cells and Msfd2a may serve as targets to modulate caveolae‐dependent transcytosis in the BBB for therapeutic purposes.

### 3.3 Restoring Efflux Transporter Activity

Efflux transporters in brain endothelial cells, mainly ABC transporters, play a key role in clearing potentially neurotoxic endogenous materials from the brain. Lower activity of efflux transporters leads to accumulation of neurotoxins in the brain, contributing to the development of neurological diseases like AD and Parkinson's disease. As such, restoring efflux transporter activity at the brain endothelial cells could be a valid therapeutic strategy.

Brain clearance of the neurotoxic A*β* peptides is partly mediated by the efflux transporters such as P‐gp, ABCC1, and ABCA1.^[^ ^69^, ^131^, ^132^ ^]^ In AD patients, A*β* clearance (efflux) from the brain is reduced by ≈30%.^[^ ^133^ ^]^ Decreased activity or function of these ABC efflux transporters could contribute to the lower efflux of A*β*. In mice, deficiency of P‐gp at the BBB increases A*β* deposition.^[^ ^134^ ^]^ In elderly non‐demented individuals, cerebral A*β* deposition has been demonstrated to be inversely correlated with P‐gp expression in the brain capillary.^[^ ^135^ ^]^ In patients with AD, reduced P‐gp function has been observed compared to healthy controls.^[^ ^136^ ^]^ Pregnane X receptor (PXR) agonist has been shown to increase P­gp expression specifically.^[^ ^104^ ^]^ Upon treatment with PXR, AD model mice showed enhanced efflux of exogenous A*β*.^[^ ^137^ ^]^ More recently, a positron emission tomography (PET)‐based method has been developed to evaluate the efficacy of drugs to induce P‐gp activity in mice,^[^ ^138^ ^]^ although this awaits clinical development. If successful, it will markedly accelerate the development of P‐gp‐targeted therapeutics.

Deficiency of ABCC1 also significantly increased A*β* deposition in the brain without affecting the production of A*β*, suggesting its involvement in the efflux of A*β* from the brain.^[^ ^105^ ^]^ Activation of ABCC1 by thiethylperazine, a drug used in the clinic to relieve nausea and vomiting, substantially reversed the cerebral accumulation of A*β* in a mouse model of AD with ABCC1 expression. Unlike P‐gp and ABCC1, ABCA1 does not directly transport A*β*.^[^ ^131^ ^]^ Instead, it affects the production and/or degradation system of A*β* by enhancing efflux of cholesterol to reduce lipidation of apoE, which is associated with increased A*β* deposition.^[^ ^69^ ^]^

## 4 BBB as a Barrier for Drug Delivery

Most drugs for brain diseases are ineffective because they fail to cross the BBB and reach diseased cells and tissues in the brain at sufficient dosages. Even under pathologically disrupted conditions, the BBB remains a formidable obstacle for the brain‐targeted delivery of therapeutics. Therefore, improvement in BBB penetration is an urgent need for the development of brain‐targeted therapeutics. Current strategies target two major areas associated with brain endothelial cells: transient disruption of the BBB tight junctions and inhibition of efflux pumps.

### 4.1 Transient Disruption of the BBB Tight Junctions

Breaking down endothelial cell junctions in the BBB is a simple and obvious strategy for brain‐targeted drug delivery. On one hand, the BBB needs to open wide enough to let therapeutics pass though, on the other hand, there is widespread concern that BBB disruption is difficult to induce without it leading to neurotoxicity. Therefore, considering the critical protective role of the BBB in the maintenance of normal brain function, such disruption requires careful management to ensure that transient opening is reversible and selective to prevent any harmful effects to the brain. Strategies using chemical and physical methods to break down the tight junctions in brain endothelial cells are under development. The pros and cons of different strategies to open the BBB are listed in **Table** **2**.

**Table 2:** Pros and cons of different strategies to open the BBB

Strategies		Methods	Pros	Cons	Ref.
Transient disruption of BBB tight junctions	Chemical methods:	Osmotic agents (mannitol)	Clinic approved. Rapid onset of action (within 5 to 10 min after administration) which results in opening the BBB to a wide range of substances	Short half‐life and side effects, such as inducing seizures and increasing intracranial pressure	^[^^139^, ^140^, ^141^, ^142^^]^
		Angiogenic molecules (VEGFA)	Approved in the clinic	Non‐specific opening of the BBB, which may cause side effects	^[^ ^35^ ^]^
		S1P	Broad effects, including regulation of brain endothelial cell junction, transcytosis, and efflux transporter activity	Dose‐sensitive	^[^^143^, ^144^^]^
		S1PR1 modulator: Fingolimod	Approved in the clinic	Size‐selective opening of the BBB, Controversial effects on BBB integrity	^[^^97^, ^145^^]^
		Autoantibodies (GRP78)	Opening of the BBB to macromolecules	Inflammation‐induced BBB breakdown may be harmful	
	Physical method:	MR‐FUS	Highly efficient, enables focus on targeted region, promising results in multiple clinical trials	Sensitive to ultrasound intensity, may cause side effects such as chronic inflammation	^[^^33^, ^146^, ^147^, ^148^^]^
Overcoming efflux transporters		Inhibitors of P‐gp and BCRP1	Some inhibitors are approved in the clinic	Mixed results in clinical studies. Potential side effects as P‐gp and BCRP1 are critical for metabolism and excretion of cytotoxic agents from the brain.	^[^^132^, ^134^, ^149^, ^150^^]^

#### 4.1.1 Chemical Methods

The BBB can be disrupted by certain chemicals. Among them, the osmotic agent mannitol is widely used in the clinic to open the BBB. Through intra‐arterial delivery, mannitol opens the BBB nonspecifically in a broad region by vasodilatation and cytoskeleton reorganization of brain endothelial cells.^[^ ^151^ ^]^ In a C6 glioma rat model, mannitol increased permeability to intravenously injected ^14^C *α*‐aminoisobutyric acid in tumor, surrounding brain tissue, and cortex by 50%, twofold, and 38‐fold, respectively. These increases were even larger in the Walker 256 carcinoma rat model.^[^ ^152^ ^]^ Similar effects by mannitol in increasing BBB permeability were also observed in patients with malignant brain tumors by as much as 50‐ to 100‐fold.^[^ ^153^ ^]^ Currently, several ongoing clinical trials are examining the effects of mannitol disruption of the BBB in brain tumor.^[^ ^139^, ^140^ ^]^ However, short half‐life and side effects, such as the potential to induce focal seizures limit its wider application.^[^ ^142^, ^154^ ^]^

Angiogenic molecules have also been used to open the BBB transiently. For instance, VEGF‐A, a classic angiogenic factor, shows strong effect in increasing BBB permeability. A low dose of systemically injected recombinant human VEGF (rhVEGF) in mice and pigs induces a short period (<4 h) of increased (three to fivefold) BBB permeability which facilitates penetration of liposomal doxorubicin to the brain (6.4‐fold increase) and significantly extends survival in a mouse model of human glioblastoma with no evidence of systemic toxicity.^[^ ^35^ ^]^ Interestingly, this method only increased permeability to nanoparticles smaller than 100 nm with no effect on nanoparticles larger than 500 nm.^[^ ^35^ ^]^ In an ischemic rat model, early postischemic (within 1 h after ischemia occurring) administration of rhVEGF165 through intravenous infusion significantly increased BBB leakage.^[^ ^155^ ^]^

Targeting the S1P‐S1PR1 signaling pathway may also be a promising method for BBB manipulation to deliver therapeutic agents into the brain. The effect of S1P on the BBB is dependent on concentration, which is regulated by the complex formed by Spns2, the major transporter for S1P and Mfsd2a in ECs.^[^ ^143^ ^]^ In this complex, Spns2 transports S1P from inside cells into the extracellular matrix with Mfsd2a improving the transport efficiency of S1P. A high concentration of S1P is required for the formation and maintenance of the BBB. Deficiency in either part of this complex decreases S1P concentration in the extracellular matrix, leading to increased BBB permeability by dysregulating both transcytosis and tight junctions. Of note, S1P replenishment reverses the low‐concentration S1P‐induced BBB breakdown. Targeting its receptor S1PR1 by genetic or pharmacological approaches (fingolimod) also facilitates a reversible and small‐molecule‐selective BBB opening (>20‐fold increase as measured by leakage of Evans blue),^[^ ^143^ ^]^ likely due to changes in the cytoskeletal association of tight junction proteins, without major signs of brain inflammation or injury in mice.^[^ ^97^ ^]^

Naturally occurring autoantibodies have also recently been shown to regulate BBB permeability.^[^ ^156^ ^]^ Neuromyelitis optica is an inflammatory disorder with prominent BBB breakdown in the acute phase of the disease. Studies of sera from neuromyelitis optica patients show reduced tight junction proteins in brain endothelial cells leading to BBB permeability.^[^ ^156^ ^]^ This is likely due to activation of NF‐*κ*B signaling pathway as studies showed that a monoclonal recombinant antibody administered to cerebral spinal fluid targets glucose‐regulated protein 78 on the surface of brain endothelial cells, leading to breakdown of tight junctions. Subsequently, the BBB becomes more permeable to endogenous macromolecules like serum albumin, immunoglobulin G, and fibrinogen. This suggests the application of these antibodies could help open the BBB transiently, providing an alternative way to deliver therapeutics to the brain.^[^ ^78^ ^]^ However, since NF‐*κ*B signaling pathway is the main pathway of inflammatory response, its modulation may be harmful in neurological diseases. Hence, the application of this method would require careful assessment and management.

#### 4.1.2 Physical Methods

Physical methods use exterior forces to open the BBB and facilitate drug delivery to the brain. A wide range of physical forces, such as ultrasound, magnetic fields, and light have been investigated to open the BBB.^[^ ^157^, ^158^, ^159^ ^]^ Among these, focused ultrasound (FUS) has gained great attention for its promising results.^[^ ^160^, ^161^, ^162^ ^]^ In the last few years, the application of FUS has been trialed in opening the BBB in glioblastoma (Identifier: NCT04446416, NCT03744026, NCT03712293, NCT04417088), AD (NCT04526262, NCT04118764, NCT03671889), amyotrophic lateral sclerosis(NCT03321487), Parkinsons disease(NCT03608553). The effect of FUS on the BBB depends on its intensity. Low‐intensity FUS activates gas‐filled microbubbles to transiently open the BBB partly by inducing an acute sterile inflammatory response in the brain.^[^ ^146^ ^]^ In a recent study, low‐intensity pulsed ultrasound opened the BBB in preclinical glioma models allowing different formulations of paclitaxel (PTX) to accumulate in the brain by three to fivefold.^[^ ^158^ ^]^ Higher intensity FUS may cause harmful chronic inflammation.^[^ ^146^ ^]^ As discussed above, employing inflammation to break down the BBB should be used with caution. Others have argued that even low‐intensity FUS also evidence neurotoxicity. Therefore, identification of the appropriate intensities of FUS and precise targeting points in the brain is essential to address neurotoxicity concerns. Magnetic resonance has therefore been introduced to guide FUS to specific target sites. It converges ultrasound to precise focal points in the brain with high accuracy over a range of intensities and frequencies.^[^ ^160^ ^]^ Recent human trials of FUS in the treatment of glioblastoma and AD in patients showed successful transient opening of the BBB with no serious adverse events being observed.^[^ ^147^, ^148^ ^]^ As in patients with AD, 95% of the targeted area by FUS shows increased BBB permeability.^[^ ^147^ ^]^

Besides FUS, a focal partial transection of the optic nerve, which is distinct from the brain, has been used to trigger a transient opening of the BBB. This method can dramatically change the biodistribution of BBB‐impermeable large albumin‐functionalized polymeric nanoparticles, increasing their accumulation in the brain by more than tenfold.^[^ ^163^ ^]^

### 4.2 Overcoming Efflux Transporter Activity

Efflux transporters at the luminal side of the brain endothelial cells can prevent drugs from gaining access to the brain. In various diseases, the activity or expression of efflux transporters is excessively high, leading to reduction in drug uptake by the brain. For example, in amyotrophic lateral sclerosis, the efflux transporter P‐gp and BCRP are both upregulated, making it more difficult for therapeutic agents to penetrate into the brain.^[^ ^164^ ^]^ Several strategies targeting efflux transporters, including modulation of expression and inhibition of activity, have been explored to improve the delivery of drugs into the brain. Activation of the A2A adenosine receptor (A2A AR) with the clinic‐approved agonist lexiscan in mice reversibly reduced expression of P‐glycoprotein and BCRP1 partly through matrix metallopeptidase 9 (MMP‐9) cleavage and ubiquitinylation, resulting in a greater accumulation of chemotherapeutic drugs in the brain. The results of this study indicate that A2A AR could be a target to improve brain‐targeted drug‐delivery.^[^ ^165^ ^]^

Activity of efflux transporters may alter without change in their expression. For example, S1P can reduce P‐gp transport activity without altering its expression. Fingolimod (FTY720), a S1P analog for the treatment of multiple sclerosis in the clinic, and its phosphorylated metabolite FTY720P rapidly reduced P‐gp activity in rat.^[^ ^144^ ^]^ P‐gp inhibitors, including cyclosporin A, PSC833, and GF120918, increased brain uptake of the anti‐tumor drug PTX in mice by three to 6.5‐fold.^[^ ^166^ ^]^ Elacridar, another P‐gp/BCRP inhibitor, sensitized amyotrophic lateral sclerosis mice to riluzole, which is normally not effective when given at the onset of symptoms.^[^ ^164^ ^]^ Since the reduced activity of P‐gp and BCRP1 on the apical side of brain endothelial cells account for A*β* accumulation in the brain parenchyma and contribute to AD pathogenesis,^[^ ^132^, ^134^, ^149^ ^]^ inhibition of expression or activity should be carefully balanced to maximize their therapeutic potential while minimizing possible side effects.

## 5 BBB as a Carrier for Drug Delivery: Harnessing Endogenous Transcytosis Systems

**Figure 4:**
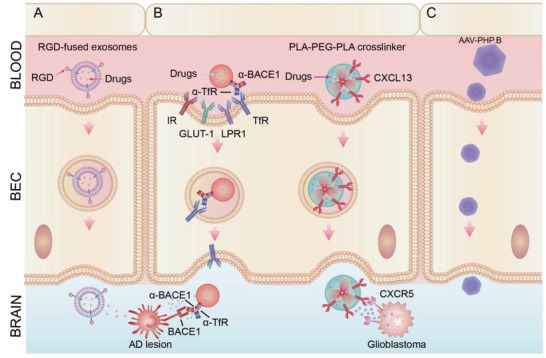
Employment of transcytosis for brain‐targeted delivery. A) Exosome‐based delivery. Exosomes that express a fusion protein of rabies virus glycoprotein (RVG) peptide, a short (29 amino acid) peptide derived from the RVG, could cross the BBB and specifically target brain cells. Systemic administration of RVG exosomes that carried siRNA against BACE1 substantially reduced levels of BACE1 mRNA expression in the brain, leading to better prognosis of AD. B) Nanoparticle‐based delivery. Engineering nanoparticles with multi‐functionalities can significantly improve the specificity and efficacy for targeted delivery. One example is a bispecific antibody, which is designed to recognize two different epitopes or antigens. A bispecific antibody targeting transferrin receptor (TfR) and BACE1 achieved superior efficacy in the treatment of AD. In the treatment of tumor in brain, Rituximab (RTX) is encapsulated within a biodegradable, crosslinked zwitterionic polymer to increase brain uptake. Further conjugating this nanocapsule with CXCL13 guided RTX nanoparticles to brain metastases of primary lymphoma to enhance the therapeutic efficacy of the antibody. C) Adeno‐associated viral (AAV) vectors are a rapidly emerging delivery platform for delivering gene and antibody‐based drugs to various cells, including neurons, astrocytes, and oligodendrocytes in the CNS and demonstrate encouraging safety and efficacy in clinical studies. AAVs enter cells via macropinocytosis, phagocytosis, clathrin, and caveolae‐mediated endocytosis. Engineered AAV capsids, AAV‐PHP.B, were recently reported to have unprecedented ability to transfer genes to the CNS in the adult mouse after systemic administration, with a >40‐fold enhancement over the previous standard AAV9, potentially transforming the ability to treat neurodegenerative diseases with AAV gene therapy.

Taking advantage of transcytosis to promote the transport of therapeutics, especially large molecules, across the BBB is a promising approach to increase their effective concentration in the brain parenchyma. Drug developers are employing different strategies to harness transcytosis for delivering a wide range of therapeutics, including nanoparticles, siRNA and mRNA, peptides, larger chemical and protein drugs, as well as therapeutic antibodies while maintaining general BBB integrity (**Figure** **4**, **Table** **3**).^[^ ^9^, ^167^, ^168^, ^169^, ^170^, ^171^, ^172^ ^]^

**Table 3:** Strategies to harness transcytosis for brain‐targeted delivery

Strategies	Method		Challenges	Ref.
Exosome‐based	Non‐functionalization	Secreted from stem cells, macrophage, monocytes, mesenchymal stromal cells, dendritic cells, and cancer cells	Limited understanding of the BBB penetration mechanism; production of sufficient quantities; increasing efficiency in loading and BBB penetration	^[^^176^, ^178^, ^179^, ^180^, ^181^, ^182^^]^
	Functionalization	Targeting specific cells: e.g., RVG exosomes		^[^^180^, ^186^^]^
Nanoparticle‐based	Single functionality	Glucose/glucose transporter GLUT1	Biocompatibility, specificity (exclusively expressed on BBB cells); efficiency (high transport capacity); minimizing interference With the natural function of receptor or carriers;	^[^ ^188^ ^]^
		Angiopep‐2/LRP1		^[^ ^189^ ^]^
		Basigin antibody/basigin		^[^ ^190^ ^]^
		CD98hc antibodies/CD98 heavy chain		^[^ ^190^ ^]^
		Transferrin/transferrin receptor		^[^^55^, ^191^, ^192^, ^193^^]^
		Dopamine receptor		^[^ ^194^ ^]^
	Multi‐functionality	Crosslinked zwitterionic polymer/CXCL13		^[^ ^195^ ^]^
		Bispecific antibody		^[^^167^, ^190^, ^196^, ^197^^]^
		Multiple therapeutic cargos (e.g., a hydrophobic drug and a hydrophilic drug)		^[^ ^198^ ^]^
Virus‐based	Naturally existed	AAV9,AAV rh.10 and rh.8	Increasing transduction efficiency in brain endothelial cells; robust safety assessment; production of sufficient quantities; reduction in autoimmunity risks	^[^ ^199^ ^]^
	Engineered	AAV‐PHP.B		^[^ ^200^ ^]^

### 5.1 Exosome‐Based Delivery

Exosomes are extracellular vehicles and small membrane‐bound carriers secreted by cells for communication with neighboring or distant cells. Exosomes are specifically derived from the endosome of cells. Exceptional intrinsic features of exosomes, including nanoscopic size (30–150 nm), favorable pharmacokinetics, low immunogenicity, and lipid‐bilayer‐enclosed structure, make them an ideal vehicle for drug delivery (Figure 4A).^[^ ^173^, ^174^ ^]^ In addition, exosomes may possess unique properties, such as specific surface proteins, to guide exosomes to specific recipient cells for binding and uptake. In the case of BBB penetration, exosomes can carry endogenous peptides and proteins from one side to the other side of the BBB.^[^ ^175^, ^176^ ^]^ A recent survey of 10 exosomes showed they all crossed the BBB, at variable rates and by a variety of mechanisms.^[^ ^177^ ^]^ With appropriate engineering, exosomes secreted from a range of cells, such as stem cells, macrophage, monocytes, mesenchymal stromal cells, dendritic cells, and cancer cells have been employed to deliver a range of therapeutic cargos to the brain parenchyma.^[^ ^176^, ^178^, ^179^, ^180^, ^181^, ^182^ ^]^ Intriguingly, in addition to the ability to penetrate the BBB, exosomes may also be involved in the maintenance of BBB integrity, as exemplified by neuron‐derived exosomes that contain miR‐132, which mediates communication between neurons and brain endothelial cells to maintain BBB integrity.^[^ ^183^ ^]^

One of the challenges in developing exosome‐based therapeutics is the production of sufficient quantities for in vivo use. Efforts have been made to prepare large quantities of exosomes. A recent study developed a cellular nanoporation biochip to stimulate cells to produce and release a larger number of exosomes (up to 50‐fold more than conventional methods). In addition, these exosomes can contain large cargos such as therapeutic mRNA, other nucleotide sequences of interest, and targeting peptides that can be delivered in quantities more than 10^3^‐fold compared to cells with low basal levels of exosome secretion.^[^ ^184^ ^]^ Although this method generated several different types of extracellular vehicles, including exosomes and microvesicles, further studies indicated more than 75% of functionally transcribed mRNAs are capsulated in exosomes.^[^ ^184^ ^]^ More recently, a synthetic biology‐inspired method has been developed to create controllable designer exosomes with dramatically increased and controllable efficiency of exosomal communication.^[^ ^185^ ^]^ In this method, a number of genes that boost exosome production, such as syndecan‐4 and NadB, have been engineered into cells to produce a 15‐ to 40‐fold increase in exosome production. Engineered exosome producing cells were subcutaneously implanted with Matrigel, a basement‐membrane matrix used for cell culture, in live mice and showed promising results in attenuating neurotoxicity and neuroinflammation in models of Parkinson's disease.^[^ ^185^ ^]^

Another major challenge in developing brain‐targeted exosome therapy is to target specific recipient cells in the brain. To improve specificity, neuron‐specific peptides have been conjugated to exosomes. One example is exosomes fused with RVG peptide, which specifically target acetylcholine receptors abundantly expressed by brain cells, such as neurons, microglia, and oligodendrocytes. RVG exosomes can efficiently cross the BBB and spread throughout the brain.^[^ ^180^, ^186^ ^]^ Systemic administration of RVG exosomes that carried siRNA against BACE1 substantially reduced levels of BACE1 mRNA expression in the brain by more than 60%.^[^ ^180^ ^]^ Similarly, in mice with focal cerebral ischemia, intravenous administration of miR‐124‐loading RVG exosomes enabled exosomes to reach the peri‐ischemic area to promote neurogenesis.^[^ ^187^ ^]^

### 5.2 Nanoparticle‐Based Delivery System

Nanoparticles are a promising tool for brain‐targeted drug delivery. Generally, 20–100 nm in at least 1D, offer a great improvement in target selectivity of a drug and overall efficacy with reduced off‐target effects and toxicity when administered systemically. An engineered nanoparticle is typically composed of a core, membrane shell, and surface ligands. Through rational design, including shape, size, and surface chemistry, nanoparticle‐based delivery systems enable more efficient delivery of a drug to its target or even directly to the diseased site.^[^ ^201^ ^]^

Although there are some common features between nanoparticles designed to restore the BBB or penetrate the BBB, distinctions in design principles reflect their central goals. Nanoparticle‐based therapeutics aimed at restoring abnormal BBB integrity only need target the luminal side of brain endothelial cells and do not necessarily need to cross the BBB. In contrast, nanoparticles targeting diseased sites in the brain parenchyma need to overcome the BBB and other barriers in the brain parenchyma. Nonetheless, the central design principle of these nanoparticles is to enhance the specificity to the target sites and minimize typical side effects caused by systemic drug delivery.

#### 5.2.1 Functionalization through Surface Modification

The current trend for brain‐targeted delivery is to design increasingly sophisticated nanoparticles with brain‐targeting ligands, which target cell surface receptors highly, or even solely, expressed on brain endothelial cells. By binding to the luminal side of brain endothelial cells, endocytosis is triggered, followed by engagement of the vesicular trafficking machinery that transports nanoparticles to the abluminal side of brain endothelial cells. Nanoparticles designed in this manner have the potential to show improved efficiency and enhanced specificity toward the brain.

##### Common Ligand‐Receptors Exploited for BBB Penetration

Common ligand‐receptor pairings utilized for the brain‐targeted delivery include transferrin/transferrin receptor, glucose/glucose transporter GLUT1, angiopep‐2/LRP1,^[^ ^168^ ^]^ as well as a number of others (Figure 4B). Transferrin receptor, a type II transmembrane protein, transports iron‐bound transferrin across the endothelium by RMT. Due to its prominent expression on brain endothelial cells, transferrin/transferrin receptor has been widely explored to increase delivery of therapeutics to the brain. Nearly all of the targeted nanoparticle‐based therapeutics that are currently in early clinical trials target transferrin/transferrin receptor.^[^ ^202^ ^]^

Glucose, the main energy source in the brain, is transported by GLUT1, which is expressed at a high level compared to many other receptors and transporters in brain endothelial cells. Enhanced GLUT1 expression can be induced by fasting with a subsequent glycemic increase, resulting in a significant increase in transport across the BBB of glucose‐conjugated nanoparticles.^[^ ^188^ ^]^

The expression of LRP1 and its ability to deliver antibody into the brain is lower than transferrin receptor and GLUT1.^[^ ^190^ ^]^ The ligand of LRP1, angiopep‐2, is a 19 aa oligopeptide that binds to LPR1 and penetrates the BBB by RMT. Angiopep‐2/LRP1 is attracting considerable interest in brain glioblastoma treatment since LRP1 is highly expressed in both brain endothelial cells, tumor cells of glioblastomas, and brain metastases of lung and skin cancers.^[^ ^189^ ^]^ Therapeutics targeting LRP1 can be guided not just through the BBB but also to tumor cells in the brain.^[^ ^203^ ^]^

Safety remains a concern when using the aforementioned receptors for brain‐targeted delivery in humans. The widespread expression of transferrin receptor and GLUT1 in peripheral organ cells limits their capability for specific brain delivery.^[^ ^196^ ^]^ The discovery of novel targets that are specific to brain endothelial cells with abundant expression might highlight new therapeutic targets. Strategies like transcriptomic and proteomic profiling have shown promising results in the identification of brain endothelial cell enriched transporters/receptors.^[^ ^12^, ^204^ ^]^ Utilizing these new technologies, extracellular MMP inducer basigin and CD98 heavy chain have been identified as highly expressed transmembrane proteins at the BBB and have shown high capacity to deliver therapeutic antibodies across the BBB.^[^ ^190^ ^]^ Furthermore, detecting antibodies capable of traversing the BBB by using in vitro or in vivo phenotyping screening with phage or yeast display libraries may also be valuable.^[^ ^190^, ^205^ ^]^

##### Ligands for Nanoparticle Functionalization

Ligands used in nanoparticle‐based brain‐targeted nanomedicine are generally natural ligands, antibodies, or peptides.^[^ ^206^ ^]^ The choice of natural ligands is somewhat limited but includes transferrin, p‐hydroxybenzoic acid, Apo‐A and E, receptor‐associated protein, glucose, insulin, and leptin. One of the disadvantages of natural ligands is that they compete with their endogenous counterparts. As an example, transferrin receptor is nearly saturated with endogenous transferrin that persists in the bloodstream at a concentration of 25 µm, meaning that a higher concentration of transferrin receptor‐targeting nanoparticle would be required to compete with the natural ligands typically found in the bloodstream.^[^ ^207^ ^]^

Antibodies that can target brain endothelial cells with high specificity would be highly desirable. Additionally, they also offer a broad selection of targets and generally do not compete with endogenous counterparts. Antibodies targeting transferrin receptor significantly improves the transport of AuNPs into the brain parenchyma without interfering with endogenous transferrin.^[^ ^192^ ^]^ Both the density (number of antibodies per nanoparticle) and affinity of antibodies conjugated to nanoparticle affect uptake by brain endothelial cells and subsequent transport across the BBB. While a high density of antibodies benefits BBB penetration, high‐affinity transferrin receptor antibody binding alters the intracellular trafficking fate of nanoparticles and hinders their transcytosis across the BBB, leading to reduced brain uptake. In comparison, lower affinity antibodies enhance uptake into the brain by facilitating dissociation from transferrin receptor.^[^ ^55^, ^191^, ^192^, ^193^ ^]^ Indeed, nanoparticles conjugated to the antibody with reduced avidity to transferrin receptor showed the greatest ability to cross the BBB. Checkpoint inhibitor antibodies to cytotoxic T‐lymphocyte‐associated antigen 4 (*α*‐CTLA‐4) and programmed cell death‐1 (*α*‐PD‐1) have shown little effect on brain glioma. This was largely due to their inability to cross the BBB. Recently, a transferrin receptor antibody has been used to assist nanoparticles containing *α*‐CTLA‐4, *α*‐PD‐1, or both to cross the BBB, leading to significantly prolonged survival in glioblastoma‐bearing mice.^[^ ^168^ ^]^ Similarly, conjugation of nanoparticles with p‐hydroxybenzoic acid, which targets dopamine receptor and promotes BBB penetration via RMT, has been used to successfully deliver *α*‐PDL1 into the brain of mice for treatment of glioma. This p‐hydroxybenzoic acid‐nanoparticle based delivery of *α*‐PDL1 prolonged survival time by reducing tumor growth more effectively in an orthotopic glioblastoma mouse model.^[^ ^194^ ^]^

One of the key disadvantages of using antibodies as ligands is that antibody production, conjugation, and subsequent purification can be extremely costly and time‐consuming.^[^ ^208^ ^]^ Peptides as targeting moieties have the advantage of ease of synthesis without compromising high specificity. Therefore, numerous synthesized peptides, such as angiopep‐2 and glutathione, have been developed to assist BBB penetration, with several candidates tested in advanced clinical trials.^[^ ^60^ ^]^ Peptides that are capable of increased uptake in the brain can be non‐selective or target specific receptors. The former belong mainly to the cell‐penetrating peptide family, but appear to be trapped in brain endothelial cells without release to the brain parenchyma.^[^ ^60^ ^]^ Therefore, development has been focused on peptides targeting brain endothelial cell receptors. Ideal candidates are those with high binding affinity and good stability to overcome multiple enzymatic barriers to reach the brain.

Identification of targeting peptides can be achieved by either extensive exploration of chemical diversity via computational means or identified by rational design from a known peptide/protein with the ability to cross the BBB. Advancements in phage display technology have helped to screen ligands with higher specificity,^[^ ^209^ ^]^ as shown by the brain‐specific phage‐derived peptide or nanoLigand Carriers that can deliver BACE1 siRNA complexes to the brain efficiently and safely.^[^ ^171^ ^]^ The length of peptides may affect the behavior of nanoparticles. Long stable positively charged peptide ligands promote natural immunoglobulin M absorption and unfavorable immunocompatibility, leading to rapid clearance from the blood and accumulation in liver and spleen. In comparison, short stable peptidomimetic ligand D8, developed by computer‐aided peptide design, successfully preserved bioactivity in the circulation and improved immunocompatibility of brain‐targeted liposomes by attenuating natural immunoglobulin M absorption.^[^ ^210^ ^]^

#### 5.2.2 Multi‐Targets Delivery

The BBB is the major but not the only obstacle for therapeutics targeting the brain to overcome. Although delivery of therapeutics across the BBB may be accomplished, low delivery to the required targeted sites in the brain parenchyma may still occur. Nanoparticles that employ multiple targets, either through engineering with multi‐functionalities or combining several therapeutics, can significantly improve the specificity and efficacy of drug delivery to the pathological site inside the brain.

##### Engineering with Multi‐Functionalities

Multi‐functional nanoparticles are also called “two‐step trojan horse.” One functionality assists nanoparticles to cross the BBB, while the other targeting ligands, typically pathological site‐specific, facilitates the specific delivery of nanoparticles to the target tissue in the brain parenchyma. For example, in the treatment of brain tumors, RTX was encapsulated within a biodegradable, crosslinked zwitterionic polymer to increase brain uptake through AMT.^[^ ^30^, ^31^ ^]^ Further conjugation of this nanocapsule with chemokine (C‐X‐C motif) ligand 13 (CXCL13), a ligand of the chemokine receptor C‐X‐C chemokine receptor type 5 (CXCR5) expressed on lymphoma, guided RTX nanoparticles to brain metastases of primary lymphoma to enhance therapeutic efficacy (Figure 4B).^[^ ^195^ ^]^

Functionality to enhance BBB penetration can also exploit RMT. One example is the bispecific antibody, which is designed to recognize two different epitopes or antigens. One epitope targets receptor a specific receptor on brain endothelial cells, such as transferrin receptor or CD98 heavy chain, to enhance BBB traversal. The other epitope recognizes the pathological target/s. Bispecific antibodies that target both BACE1 and transferrin receptor achieved superior efficacy in reducing brain A*β* levels with minimal sustained toxicity in both rodent and primate AD models.^[^ ^167^, ^190^, ^196^ ^]^ In glioblastoma, bispecific antibody targeting VEGFA and angiopep‐2 show highly effective inhibition of tumor growth through vascular and/or immunomodulatory effects.^[^ ^211^ ^]^

##### Combination Therapies

Combination therapies where several therapeutics are used simultaneously are becoming increasingly popular in drug development, due to increased therapeutic effects and reduction in toxicity, especially for cancer treatment. There are more than 10 000 ongoing clinical trials currently registered in the US alone investigating combination therapies for different diseases.^[^ ^212^ ^]^ Nanoparticles can readily be designed to accommodate multiple therapeutic cargos which are sequentially or synchronously released, leading to highly controlled drug delivery compared to conventional targeting strategies. A proof‐of‐concept study showed that transferrin‐functionalized polyethylene glycol‐modified liposome nanoparticles (LNPs) can be used to deliver combination therapies across the BBB for the treatment of glioblastoma. These nanoparticles contained a hydrophobic drug (bromodomain inhibitor JQ‐1) in the lipid envelope while incorporating a hydrophilic drug (temozolomide) in the inner core. When the nanoparticles reach the targeted sites in tumor, the outer layer of the particle degrades, releasing the bromodomain inhibitor JQ‐1. ≈24 h later, temozolomide is released from the particle core.^[^ ^198^ ^]^

A large percentage of glioblastomas are resistant to temozolomide due to the coactivation of the epidermal growth factor receptor (EGFR) family and receptor tyrosine kinase proteins, including mesenchymal‐epithelial transition factor (MET). To address this chemoresistance, inherbin3 (denoted as EGFR‐binding peptide, EBP) and cMBP (denoted as MET‐binding peptide, MBP) were conjugated on the surface of NHS‐PEG8‐Mal modified 2‐methacryloyloxyethyl phosphorylcholine (MPC) to form a novel dual functionalized brain‐targeting nanoinhibitor, BIP‐MPC‐NP.^[^ ^197^ ^]^ Compared with BIP‐NPs (without MPC), BIP‐MPC‐NPs achieved 3.2‐fold increase in brain uptake. In terms of anti‐glioblastoma effect, tumor volume was greatly reduced (12.6 mm^3^ after 21 days of treatment with BIP‐MPC‐NPs) compared to temozolomide‐treated mice (105.1 mm^3^ after 21 days of treatment) representing an ≈90% decrease in tumor size. In addition, mice treated with EBP‐ or MBP‐MPC‐NP for 21 days showed 75% reduction in tumor volume to 50 mm^3^, further demonstrating the enhanced anti‐tumor effect of combination therapy.

### 5.3 Virus‐Based Delivery

AAV vectors are a rapidly emerging delivery platform for delivering gene and antibody‐based drugs to various cells, including neurons, astrocytes, and oligodendrocytes in the brain and demonstrate encouraging safety and efficacy in clinical studies (Figure 4C).^[^ ^199^ ^]^ AAVs enter cells primarily through macropinocytosis, phagocytosis, clathrin, or caveolae‐mediated endocytosis. Notably, AAV vectors provide a powerful tool for gene replacement or silencing to address loss‐of‐function and gain‐of‐function mutations, respectively, since they exhibit durable pharmacology. These attributes suggest benefits for neurological disorders, which often require complex therapeutic strategies. More than 100 natural AAV variants have been identified in humans, non‐human primates, and other vertebrates.^[^ ^213^, ^214^ ^]^ Among them, AAV9 is the first one to demonstrate the ability to cross the BBB and to enable transgene delivery to the brain.^[^ ^215^ ^]^ Several other naturally occurring AAV vectors, including rh.10 and rh.8, were also found to enter the brain in both neonatal and adult mice. Engineered AAV capsids were recently reported to have unprecedented ability to transfer genes in the brain in adult mice after systemic administration, with a >40‐fold enhancement over the previous standard AAV9.^[^ ^200^ ^]^

## 6 Neuroinflammation and the BBB

Under normal physiological conditions, a low level of immune cell trafficking across the BBB occurs as a normal component of the immunosurveillance of the brain, which is required for effective immunity in the brain and infectious pathogen clearance. In many diseases of the brain, such as AD, amyotrophic lateral sclerosis, Parkinson's disease, and multiple sclerosis, neuroinflammation‐induced BBB permeability creates a route of access for circulating pathogens. When inflammation is present, an altered BBB breaks the immune privilege of the brain, exposing neuronal antigens to the peripheral inflammatory molecules, which further stimulate the inflammatory response in the brain, leading to accelerated neurological disease development.^[^ ^216^, ^217^, ^218^, ^219^ ^]^ As such, communication between the immune system and the brain has become recognized as a central element of healthy brain function.^[^ ^220^ ^]^

On one hand, targeting inflammation restores BBB integrity, which in turn benefits the treatment of neurological diseases associated with neuroinflammation. On the other hand, infiltration of immune cells to the brain parenchyma during neuroinflammation presents a great opportunity for therapeutics to cross the BBB. To this end, we focus on neuroinflammation to demonstrate new strategies to restore BBB integrity and take advantage of the carrier property of BBB to develop brain‐targeted therapeutics.

**Figure 5:**
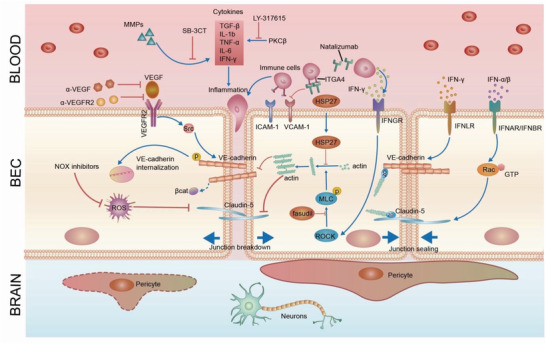
Targeting inflammation to restore or penetrate the BBB. Targeting inflammation and downstream sequalae, including angiogenesis, oxidative stress, or cytoskeleton reorganization, restores the BBB. Pharmacological inhibitors of inflammatory cytokines rescue damage to the BBB. Inhibitors of angiogenesis (e.g., antibodies targeting VEGF/VEGFR) and oxidative stress (inhibitor to NOXs) restore BBB integrity. Targeting immune cells is an alternative way to inhibit inflammation. Natalizumab, which targets the *α*4 integrin (ITGA4), prevents the recruitment of immune cells to brain endothelial cells by inhibiting the integrin‐VCAM1 interaction and greatly rescues BBB integrity. IFN‐*α*/*β* enhances BBB integrity via activation of Rac‐1. IFN‐*λ* tightens the BBB through restricting IFNLR1 in the BBB. Cytoskeleton reorganization is downstream effect of inflammation and disrupts the BBB through actin polymerization. Fasudil (ROCK inhibitor), Heat shock protein 27 (HSP27), LY‐317615 (PKC*β* inhibitor) all restore the BBB by re‐normalizing the cytoskeleton.

**Figure 6:**
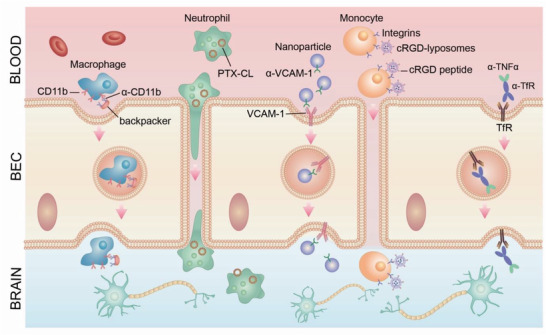
Bioinspired brain‐targeted delivery strategies. Immune cells can be used as a “trojan horse” for brain‐targeted delivery. Therapeutics can be either encapsulated in cells, conjugated to the cell surface or utilize “backpacks.” Conjugating LNPs with ligands of VCAM‐1 significantly increase accumulation in the inflamed brain endothelium. The anticancer drug PTX was first encapsulated into cationic liposomes (PTX‐CL), followed by merger with NEs to form PTX‐CL/NEs to migrate across the BBB and target inflamed brain where infiltrating tumor cells locate. cRGD liposomes co‐migrate with monocytes to cross BBB. Therapeutics containing “backpacks” attach to macrophages to cross the BBB. Alternatively, bispecific antibodies which target both brain endothelial cells and inflammatory cytokine in the brain parenchyma show potential to inhibit neuroinflammation. Chronic treatment with the cTfRMAb‐TNFR fusion protein, a BBB‐penetrating biologic TNF‐*α* inhibitors, offers therapeutic benefits by targeting neuroinflammation, leading to the restoration of BBB integrity.

In this part, we review recent progress in strategies to target inflammation in the brain from two perspectives: 1) to restore the BBB integrity by targeting inflammation in the brain vasculature (**Figure** **5**); 2) to learn from immune cell brain invasion to deliver therapeutics across the BBB (**Figure** **6**).

### 6.1 Targeting Inflammation to Restore the BBB

Inflammation provides therapeutic opportunities for neuronal protection against brain diseases. Indeed, anti‐inflammation agents have already been used in the clinic to treat neurological diseases with disrupted BBB. For example, glucocorticosteroids that have anti‐inflammatory and immunosuppressive effects are used to control unwanted inflammatory response and improve BBB integrity in multiple sclerosis patients.^[^ ^221^ ^]^ Therapeutic agents targeting inflammation and its downstream signaling pathways, such as angiogenesis, oxidative stress, and cytoskeleton reorganization have also been explored to restore the BBB (Figure 5; **Table** **4**).

**Table 4:** Targeting inflammation and relevant signaling pathways to restore the BBB

Targets		Diseases	Therapeutics	Ref.
Cytokines	TGF‐*β*	Cognitive impairments	TGF‐*β* inhibitor	^[^ ^217^ ^]^
	IL‐6	Ischemia	Anti–IL‐6 antibody	^[^ ^220^ ^]^
	IL‐1*β*	Seizure syndrome	IL‐1*β* receptor antagonists: Anakinra	^[^^224^, ^225^^]^
	TNF*α*	Depression	TNF*α* inhibitor: etanercept	^[^ ^226^ ^]^
	Prekallikrein	Multiple sclerosis	KK antibody	^[^ ^229^ ^]^
	PKC*β*	Multiple sclerosis	LY‐317615	^[^ ^231^ ^]^
Adhesion molecules/ligands	Integrin	Multiple sclerosis	Natalizumab	^[^^237^, ^238^^]^
	ICAM‐1	Ischemic stroke	Enlimomab	^[^ ^239^ ^]^
Angiogenic factor	VEGF‐A	Amyotrophic lateral sclerosis, Alzheimer's disease, Parkinson's disease, epilepsy, ischemic stroke	VEGF‐A	^[^^155^, ^242^^]^
Oxidative stress	NOX4	Cerebral ischemia‐reperfusion	GKT136901	^[^ ^253^ ^]^
	NOX5	Cerebral ischemia‐reperfusion	ML090	^[^ ^254^ ^]^
	MMP9	Schizophrenia	SB‐3CT	^[^ ^257^ ^]^
Cytoskeleton	RohA/ROCK signaling	Cerebral cavernous malformation	Fasudil	^[^^258^, ^259^^]^
	Actin polymerization	Ischemia/reperfusion stroke	HSP27	^[^ ^260^ ^]^
	Annexin A1(ANXA1)	Multiple sclerosis	Glucocorticosteroids	^[^ ^261^ ^]^

#### 6.1.1 Inflammatory Cytokines

Inflammatory cytokines, such as transforming growth factor‐*β* (TGF‐*β*), interleukin‐1 *β* (IL‐1*β*), tumor necrosis factor alpha (TNF‐*α*), and IL‐6, have been shown to be upregulated with the onset of pathologic processes characterized by compromised BBB function.^[^ ^21^ ^]^ TGF‐*β* is a critical mediator of neuroinflammation.^[^ ^222^ ^]^ On one hand, TGF‐*β* increases the paracellular permeability of vascular endothelial monolayers through tyrosine phosphorylation of VE‐cadherin and claudin‐5.^[^ ^223^ ^]^ On the other hand, BBB breakdown further triggered TGF‐*β* signaling in astrocytes, leading to cognitive impairments in aging rodents. Pharmacological inhibition of TGF‐*β* signaling reversed these symptomatic outcomes in aged mice.^[^ ^217^ ^]^ Several lines of evidence from animal studies also support a role for IL‐1*β* in BBB dysfunction during neuroinflammation. Treatment of IL‐1*β* receptor antagonists or genetic deletion of the IL‐1 receptor attenuates BBB hyperpermeability induced by neuroinflammation in mice.^[^ ^224^ ^]^ Anakinra, an analog of IL‐1 receptor antagonist is the only effective treatment for a seizure syndrome induced by infection.^[^ ^58^, ^225^ ^]^

In a mouse model of depression, TNF*α*‐induced NF‐*κ*B signaling, a classic inflammation activator and repressed claudin‐5 expression in mouse brain endothelial cells. Treatment with the TNF*α* inhibitor, etanercept, reduced hippocampal BBB permeability in depressed mice.^[^ ^226^ ^]^ Likewise, melatonin decreased lipopolysaccharide‐induced BBB damage by activating AMP‐activated protein kinase, leading to reduced tight junction degradation and rescue of BBB function.^[^ ^227^ ^]^ Anti–IL‐6 neutralizing antibody was shown to modulate BBB integrity in the ovine fetus.^[^ ^220^ ^]^ The kallikrein (KK)‐kinin system has been shown to open the endothelial barrier in acute inflammation.^[^ ^228^ ^]^ Plasma prekallikrein, the precursor of KK, was markedly upregulated in active brain lesions of multiple sclerosis patients. In the experimental autoimmune encephalomyelitis (EAE) mouse model, a model of brain inflammatory disease, KK antibody applied after the onset of neurological symptom significantly attenuated the signs of EAE in mice with a remarkable reduction of BBB disruption and brain inflammation.^[^ ^229^ ^]^ In addition, activation of protein kinase C*β* (PKC*β*) augmented expression of a broad range of inflammatory mediators,^[^ ^230^ ^]^ while inhibition of PKC*β* by LY‐317615 stabilized the BBB in EAE mice.^[^ ^231^ ^]^

IFNs are another major family of antiviral cytokines.^[^ ^232^ ^]^ There are three types of IFNs. The type I IFNs consist of the ligands IFN‐*α* and IFN‐*β* subtypes. Unlike some inflammatory stimuli that increase BBB permeability, IFN‐*α*/*β* enhances BBB integrity via activation of Ras‐related C3 botulinum toxin substrate 1 (Rac‐1).^[^ ^103^ ^]^ Activation of TAM receptors, Mertk, synergized with IFN‐*β* to tighten cell junctions and prevent virus transit across brain endothelial cells, suggests a new therapeutic application for TAM antagonists that are currently in clinical development.^[^ ^28^ ^]^ The type II IFN family consists solely of IFN‐*γ*, a pro‐inflammatory cytokine. In the context of brain infections, IFN‐*γ* disrupts the BBB through both direct and indirect effects. Direct effects include down‐regulation and/or internalization of tight junction proteins, whereas indirect pathways consist of enhancing leukocyte trafficking and promoting the expression of other inflammatory cytokines and chemokines.^[^ ^233^, ^234^ ^]^ IFN‐*λ*, also known as type III IFN, tightens the BBB and restricts viral neuroinvasion and pathogenesis through restricting IFNLR1 in the BBB. This makes IFN‐*λ* an exciting potential therapeutic option for neuroinvasive infections and other diseases that involve a breakdown of the BBB, including brain autoimmune disorders.^[^ ^235^ ^]^

Another strategy to target inflammation in the BBB is to focus on inducible cell‐adhesion molecules on the surface of brain endothelial cells. Compared with peripheral endothelial cells, healthy brain endothelial cells exhibit lower expression of leukocyte adhesion molecules such as vascular cell adhesion protein 1 (VCAM‐1) and intercellular adhesion molecule 1 (ICAM‐1), which aid in maintaining low levels of immune surveillance in the brain. Inflammation upregulates expression of adhesion molecules to initiate binding of leukocytes, the first step of leukocyte entrance into brain tissues. VCAM‐1 up‐regulation at the BBB is a crucial step in age‐related cognitive deficits, indicating VCAM‐1 as a potential therapeutic target for age‐related neurodegeneration.^[^ ^236^ ^]^ Leukocytes bind to endothelial cells through integrin‐VCAM‐1 interaction. Targeting integrin, expressed by leukocytes, is an alternative way to inhibit inflammation. Natalizumab, which targets the *α*4 integrin, inhibits the interaction between *α*4 integrin and VCAM‐1, thus preventing recruitment of immune cells to brain endothelial cells. In turn, it greatly rescues BBB integrity and reduces new lesion formation in relapsed multiple sclerosis.^[^ ^237^, ^238^ ^]^ To date, targeting ICAM‐1 has generated mixed results. A murine antibody to human ICAM‐1 (enlimomab), produced significant protective effects in animal studies, however, showed no significant improvements in humans, likely due to the detrimental immunoactivation of anti‐mouse antibodies.^[^ ^239^, ^240^ ^]^

Inflammation‐mediated BBB disruption may also occur through the activation of microglia and astrocytes.^[^ ^241^ ^]^ With direct physical contact with brain endothelial cells, vessel‐associated microglia support BBB integrity via maintaining the expression of the tight‐junction protein claudin‐5. Sustained inflammation induces migration of brain resident microglia to the brain vasculature. As a result, microglia phagocytose astrocytic end‐feet and impair BBB function.^[^ ^241^ ^]^

#### 6.1.2 Angiogenesis

Angiogenesis is associated with BBB permeability in many neurological diseases.^[^ ^242^ ^]^ However, the effect of angiogenesis in neurological diseases is mixed. For example, VEGF‐A is the best‐known angiogenic agent and a potent BBB permeability inducer. However, low levels of VEGF‐A are necessary for endothelial cell survival and for maintaining BBB integrity.^[^ ^243^ ^]^ Under pathological conditions such as inflammation and hypoxia, high level of VEGF‐A destroys the BBB partly through modulating claudin‐5, leading to advanced stages of neuroinflammatory diseases.^[^ ^155^, ^244^ ^]^ However, in other instances angiogenesis may be beneficial to brain vasculature and function. As an example, antibodies against Nogo‐A, a neurite outgrowth inhibitor and an angiogenesis inhibitor in the brain,^[^ ^245^ ^]^ improve vascular sprouting and reduces neurological deficits after cerebral ischemia in mice.^[^ ^246^ ^]^ These findings were reproduced in a clinical setting where anti–Nogo‐A antibodies were administered intrathecally for spinal cord injury.^[^ ^246^ ^]^

#### 6.1.3 Oxidative Stress

Oxidative stress appears to be a key link between inflammation and angiogenesis, contributing to BBB disruption in cerebral vascular diseases, such as stroke and traumatic brain injury and other neurological disorders including AD and Parkinson's disease.^[^ ^247^, ^248^, ^249^, ^250^, ^251^ ^]^

Redox balance is important for the maintenance of brain function. However, uncontrolled oxidative stress, often presenting as a chronic imbalance between cellular pro‐oxidants and antioxidants, causes toxic reactive free radicals and breakdown of BBB tight junctions.^[^ ^249^, ^252^ ^]^

Upon cerebral ischemia‐reperfusion, intracellular calcium increases, leading to the activation of NADPH oxidase 4(NOX4)^[^ ^253^ ^]^ and NOX5,^[^ ^254^ ^]^ two primary sources of oxidative stress. These further promote BBB breakdown, leading to the entry of inflammatory cells and other toxins that cause neuronal cell death and progressive brain injury. Drugs that selectively inhibit NOX4 (GKT136901) and NOX5(ML090) reduced BBB permeability, resulting in a significant reduction of infarct volume and direct neuroprotection when given immediately before or at the time of reoxygenation in a mouse model of cerebral ischemia‐reperfusion.^[^ ^253^, ^254^ ^]^

Oxidative stress also induces microglial activation and redox‐sensitive MMP stimulation, especially MMP2 and MMP9, leading to activation of inflammatory signaling pathways, such as NF‐*κ*B and secretion of various cytokines that ultimately opens the BBB to facilitate leukocyte migration across the BBB.^[^ ^255^, ^256^ ^]^ In schizophrenia, blocking MMP9 activation by the specific MMP2/9 inhibitor SB‐3CT breaks the interaction between oxidative stress and neuroinflammation, leading to accelerated recovery.^[^ ^257^ ^]^

#### 6.1.4 Cytoskeleton Reorganization

Cytoskeleton reorganization is one of the downstream consequences of inflammation. The stability of brain endothelial cell junctions is maintained by anchoring tight junction and adherens junction proteins to the actin cytoskeleton via multiple accessory proteins such as ZOs. In pathological conditions such as ischemia stroke, the actin filaments polymerize into linear stress fibers. The actin–myosin cytoskeleton contracts via myosin light chain phosphorylation, leading to increased cytoskeletal tension, disassembly of junction proteins, and widening of the paracellular space between brain endothelial cells and eventually increased BBB permeability.^[^ ^262^ ^]^ One of the key modulators of the cytoskeleton is small Rho GTPases, including the RhoA and Rac‐1.^[^ ^263^ ^]^ Activation of the RhoA/Rho‐associated protein kinase (ROCK) signaling pathway leads to tight junction protein phosphorylation and internalization, followed by BBB breakdown. A ROCK inhibitor, fasudil, reduces BBB disruption in ischemia and cerebral cavernous malformation.^[^ ^258^, ^259^ ^]^ Another example is heat shock protein 27 (HSP27), a direct inhibitor of actin polymerization. In mice, intravenous administration of a cell membrane‐permeable HSP27 protein after post‐ischemic reperfusion rapidly elevated HSP27 levels in the BBB, leading to amelioration of ischemia/reperfusion stroke‐induced BBB disruption and subsequent neurological deficits.^[^ ^260^ ^]^ The effect of glucocorticosteroid is partly mediated through its downstream target annexin A1(ANXA1), which regulates paracellular permeability in the BBB via interaction with the actin cytoskeleton and down‐regulation of RhoA GTPase activity. In multiple sclerosis patients, ANXA1 is lost. Treatment of brain endothelial cells with recombinant ANXA1 restores cytoskeleton architecture and reduces paracellular permeability.^[^ ^261^ ^]^

### 6.2 Bioinspired Brain‐Targeted Delivery: Learning from Inflammation

The brain is generally considered to have a low number of immune cells derived from the blood‐stream due to the presence of the BBB. When the BBB is compromised by neuroinflammation, leukocytes are extensively recruited into the brain parenchyma.^[^ ^264^, ^265^ ^]^ This passage across the BBB is not due to BBB paracellular permeability, which would also permit the passage of much smaller and more numerous erythrocytes, thereby inducing hemorrhage. Instead, the entry of immune cells under normal and inflammatory conditions is by the highly orchestrated process of diapedesis.^[^ ^56^, ^57^, ^58^ ^]^ The recruitment is achieved in part through leukocyte‐derived cytokines activating the brain endothelial cells, inducing expression of adhesion molecules, such as ICAM‐1 and VCAM‐1, which mediate leukocyte recruitment. These immune cells, migrate across the BBB through either chemotaxis or diapedesis, offer an opportunity to deliver therapeutics to the brain as a “trojan horse” (Figure 6).

#### 6.2.1 Inflammation‐Targeted Delivery to Restore the BBB

Targeting inflammation systemically or non‐specifically may have significant potential to compromise host defense against infections. Inflammation‐targeted nanomedicine may be able to attenuate these risks due to enhanced selectivity.

Inflammation induces ICAM‐1 and VCAM‐1 specifically on the luminal side of brain endothelial cells, making these adhesion molecules easily accessible to nanoparticles. Indeed, these have been used to guide nanoparticles to inflammation sites in the BBB at sufficient concentration. Nanoparticles coated with antibodies against ICAM‐1 experience 100‐fold higher uptake than uncoated nanoparticles in the inflamed brain.^[^ ^266^ ^]^ An interesting recent study further showed that conjugating LNPs with ligands to VCAM‐1 provides even higher (≈eightfold) cerebral accumulation than ICAM‐1/LNP in the inflamed brain endothelium.^[^ ^267^ ^]^

Resolvin D (RvD) derived from docosahexaenoic acid decreases the leukocyte–endothelial cell interaction, leading to inhibition of inflammation. Neutrophil membrane‐derived nanoparticles loaded with RvD were able to specifically bind to the inflamed endothelium in mouse brain with ischemia/reperfusion stroke, leading to inhibition of inflammation, the rescue of BBB integrity, and prevention of neurological damage during reperfusion.^[^ ^268^ ^]^

Thrombomodulin is an endothelial surface glycoprotein that protects endothelial barrier function from inflammation. Loading nanoparticles with thrombomodulin mRNA induces de novo expression of thrombomodulin, leading to inhibition of TNF*α*‐induced BBB permeability and alleviation of cerebrovascular edema.^[^ ^267^ ^]^

#### 6.2.2 Inflammation‐Inspired Delivery to Cross the BBB

The process that immune cells use to cross the BBB can be employed to deliver therapeutics into the brain. Therapeutic cargos can be either internalized or attached to surface of immune cells. So far, a number of immune cells, including neutrophils, monocytes, and macrophages have been used as a “trojan horses” for brain‐targeted delivery.

Macrophages have been used to deliver the nanoformulated redox enzyme, catalase, to mitigate oxidative stress, leading to prolonged survival of dopaminergic neurons in animal models of

Parkinson's disease.^[^ ^269^ ^]^ The best nanoenzymes for delivery in macrophages have a relatively large size (≈200 nm) which results in improved loading capacity and release from macrophages. In contrast, small‐sized nanoparticles require a polyethylene glycol corona for stealth properties and to avoid entrapment in monocytes and macrophages. To maximize the loading of therapeutics, a cell “backpack” approach has been developed. The “backpacks” consist of multiple micropatches made from a few hundred nanometer thick polymer layers (e.g., >50 layers) that adhere to the surface of immune cells. These polymer layers often consist of protective/structural layers, release layers, and drug‐encapsulating layers in the middle. Backpacks conjugate to macrophages through CD11b antibodies and have been successfully used to deliver therapeutics to the inflamed brain.^[^ ^270^ ^]^

Neutrophils are highly enriched in solid tumor, neuroinflammatory and neurodegenerative diseases, contributing to diseases progression.^[^ ^271^, ^272^ ^]^ Due to their ability to cross biological barriers including the BBB, they have been used to carry liposomes that contain anti‐cancer drug to treat glioblastoma.^[^ ^273^ ^]^ PTX was first encapsulated into cationic liposomes (PTX‐CL) and then merged with neutrophils to form PTX‐CL/neutrophils complex. Surgery to remove tumor tissues in the brain results in local brain inflammation, which releases inflammatory factors that promote PTX‐CL/neutrophil migration across the BBB and trigger the release of PTX‐CL from the neutrophils to inhibit tumor recurrence.^[^ ^273^ ^]^ In this work, PTX‐CL was internalized into neutrophils through incubation. An alternative approach enables cargo loading into neutrophils after administration. For instance, drug‐loaded albumin nanoparticles (nanoparticles made from denatured bovine serum albumin) have been shown to be specifically internalized by neutrophils activated in situ, which in turn, migrate across blood vessels into inflammatory tissues.^[^ ^274^ ^]^ Encapsulation of core‐shell structured magnetic mesoporous silica nanoparticles into neutrophils has recently been used as tracking probes or drug delivery nanocarriers for inflamed glioma‐targeting theranostics.^[^ ^275^ ^]^

Besides internalization in immune cells, nanoparticles can also be attached to the surface through ligand‐receptor interaction. Peptide‐modified nanoparticles with RGD sequence (Arg‐Gly‐Asp), specifically bind to the integrin *α*v*β*1 receptors that are highly expressed on the surface of leukocytes and show robust brain uptake in response to inflammation. Further modification of RGD to cyclic RGD peptide (cRGD, Arg‐Gly‐Asp‐D‐Phe‐Lys) generates an even higher affinity for integrin receptors on monocytes. This approach was exploited to deliver anti‐depression drug‐containing cRGD liposomes, which co‐migrate with monocytes or neutrophils, to cross BBB, leading to significant rescue of depression‐like behavior or ischemic stroke in mice.^[^ ^276^, ^277^ ^]^

Instead of using whole intact immune cells, cloaking the nanoparticle surface with membrane fragments derived from leukocyte or macrophages “disguise” the nanoparticles, enabling these to behave as immune cells, prolonging their retention in the circulation and enabling them to utilize existing extracellular molecular machinery of immune cells to bypass the BBB and target pathological sites.^[^ ^278^, ^279^ ^]^

Another approach that uses bispecific antibodies that target both brain endothelial cells and inflammatory cytokines in the brain parenchyma shows potential to inhibit neuroinflammation. Chronic treatment with the cTfRMAb‐TNFR fusion protein, a transferrin receptor mediated BBB‐penetrating biologic TNF‐*α* inhibitor, reduces neuroinflammation, leading to the restoration of BBB integrity and overall improving cognitive function in a mouse model of AD.^[^ ^280^ ^]^

## 7 Challenges and Future Direction

The BBB field has so far made significant progress in identifying key molecules that mediate BBB function. However, despite decades of effort in developing BBB‐targeted therapy for the treatment of animal models of various diseases, it is important to note there is still limited success in the translation of these therapies into the clinic especially for serious brain diseases.^[^ ^202^ ^]^ Several important questions remain regarding BBB function in the context of disease and how this barrier can be safely targeted.

The first focus is the inherent properties of the BBB. How are BBB function and integrity regulated? Are alterations in the BBB induced by the same or different signals across various neurological conditions? The BBB is a highly active complex constantly changing between an “open” and “closed” state to transport nutrients but block toxins. Considering the regional heterogeneity across the vascular tree in the brain, some brain regions may be more vulnerable to certain disease pathologies than others. It is critical to understand exactly how the complex physiology of the BBB is regulated in various brain regions under different pathological conditions. This will help to determine whether a particular strategy will be applicable to a wide range of disorders or specific strategy is required for each disease. Transcriptomic analyses, especially single‐cell RNA sequencing, and proteomic profiling may reveal the mechanistic differences in various parts of the BBB and provide therapeutic targets for BBB repair in a range of neurological conditions.

What are the relationships between dysfunctional BBB and pathology in the brain parenchyma? Many diseases in the brain have dysfunction in both neurology and the BBB, suggesting a close link between neurological diseases and BBB breakdown.^[^ ^74^ ^]^ However, we still have little idea whether a dysfunctional BBB is a cause or consequence of the neurological disorder. It is important to note that clinical trials aiming to repair the BBB by reducing brain inflammation or targeting brain A*β* in AD patients have so far failed.^[^ ^281^, ^282^ ^]^ Combination therapies that target both dysfunctional BBB and pathological sites inside the brain parenchyma may provide a better way to treat these diseases. To this end, understanding the origin of each specific neurological disease would lead to better identification of tractable therapeutic targets.

How to improve specificity in targeting the brain? There are two levels of specificity. The first level is the specificity toward the brain. Local administration is generally more specific than systematical administration. However, local administration is often too invasive and may promote local inflammation. The second level of specificity is targeting pathological brain sites over normal regions in the brain.^[^ ^267^, ^283^ ^]^ Therapeutics with multifunctionalities have shown promising results in guiding therapeutics to diseased sites, either in the brain vasculature or inside the brain parenchyma. However, inadequate specificity and efficacy toward diseased brain regions are still major hurdles to clinical application. Considerable efforts have so far been made to identify high‐affinity ligands and better understand ligand‐receptor interaction of the human BBB. The discovery of specific receptors on the BBB and the exclusive pathological markers are key for specific and enhanced targeting of the abnormal brain.

The second focus is on the properties of therapeutics. When administered systematically, therapeutics may not be able to maintain stability in the circulation resulting in premature payload release, potentially leading to systemic drug toxicity and adverse off‐target tissue effects. In the case of nanoparticle‐based nanomedicine, several lines of evidence suggest nanoparticle‐associated toxicity, including harmful interaction with vascular system and neurons, may hinder their further application.^[^ ^284^ ^]^ Therefore, a comprehensive and quantitative understanding of the behavior of nanoparticles in the body, at the molecular level, is important to precisely control transport and clearance of nanoparticles and minimize the potential health hazards of nanomedicines. There is still a lack of understanding of the physicochemical properties of nanoparticles that determine their ability to cross the BBB. Transcytosis and tight junction in brain endothelial cells play a central role in transporting nanoparticles across the BBB,^[^ ^15^, ^198^, ^285^, ^286^ ^]^ but current technologies are not able to provide information on the dynamic vesicular transport and junction change in real‐time in live animals, due to poor resolution and limited penetration into brain tissues. Novel imaging systems, which are less invasive but offer greatly enhanced spatial resolution, are required. Such systems will allow precise tracking, with unprecedented fidelity, of the journey of nanoparticles across the BBB.^[^ ^287^ ^]^ Ultimately, these insights may provide novel design principles for nanoparticle‐based delivery system to maximize the benefits for current brain‐targeted therapeutics.

## Conflict of Interest

The authors declare no conflict of interest.
